# Doping-enhanced radiative efficiency enables lasing in unpassivated GaAs nanowires

**DOI:** 10.1038/ncomms11927

**Published:** 2016-06-17

**Authors:** Tim Burgess, Dhruv Saxena, Sudha Mokkapati, Zhe Li, Christopher R. Hall, Jeffrey A. Davis, Yuda Wang, Leigh M. Smith, Lan Fu, Philippe Caroff, Hark Hoe Tan, Chennupati Jagadish

**Affiliations:** 1 Department of Electronic Materials Engineering, Research School of Physics and Engineering, The Australian National University, Canberra, Australian Capital Territory 2601, Australia; 2 Centre for Quantum and Optical Science, Swinburne University of Technology, Hawthorn, Victoria 3122, Australia; 3 Department of Physics, University of Cincinnati, Cincinnati, Ohio 45221-0011, USA

## Abstract

Nanolasers hold promise for applications including integrated photonics, on-chip optical interconnects and optical sensing. Key to the realization of current cavity designs is the use of nanomaterials combining high gain with high radiative efficiency. Until now, efforts to enhance the performance of semiconductor nanomaterials have focused on reducing the rate of non-radiative recombination through improvements to material quality and complex passivation schemes. Here we employ controlled impurity doping to increase the rate of radiative recombination. This unique approach enables us to improve the radiative efficiency of unpassivated GaAs nanowires by a factor of several hundred times while also increasing differential gain and reducing the transparency carrier density. In this way, we demonstrate lasing from a nanomaterial that combines high radiative efficiency with a picosecond carrier lifetime ready for high speed applications.

## Abstract (web summary)

Until now, efforts to enhance the performance of nanolasers have focused on reducing the rate of non-radiative recombination. Here, Burgess *et al.* employ controlled impurity doping to increase the rate of radiative recombination.

## Graphical abstract

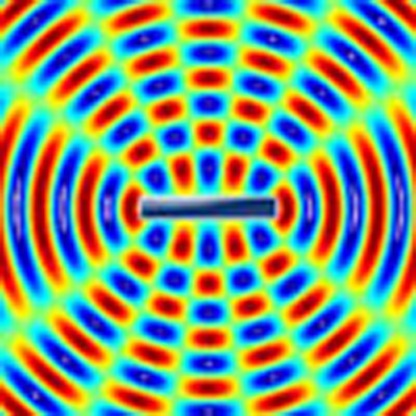

Combining both cavity and gain medium, the semiconductor nanowire (NW) has proven a popular and versatile platform for realizing nanolasers. Published examples now include photonic^1^,^2^,^3^,^4^,^5^,^6^,^7^,^8^,^9^,^10^,^11^,^12^,^13^,^14^,^15^,^16^,^17^,^18^,^19^,^20^, plasmonic^21^,^22^,^23^,^24^,^25^ and polaritonic^26^ devices operating across wavelengths from the infrared to ultraviolet. A particular advantage of the NW geometry is its amenability to the fabrication of heterostructure, enabling nanolasers that employ complex band and strain engineering^6^,^14^,^18^,^20^ as well as the heterogeneous growth of nanolasers on substrates such as silicon^9^,^14^,^20^. This hybridization of NW lasers with planar silicon structures holds potential for delivering novel integrated photonic systems^22^ and has generated particular interest in the development of near-infrared NW nanolasers. To date, room temperature, near-infrared operation has been demonstrated from commercially significant III–V materials such as GaAs^7^,^10^,^11^,^17^,^25^, InGaAs^9^,^15^,^18^ and InP^12^,^16^,^19^ nanolasers.

In all cases, material quality has been found to be of critical importance for room temperature operation with authors working to minimize the defect^20^,^26^ and surface state density^10^,^15^ to minimize the rate of non-radiative recombination. For materials exhibiting a high surface recombination velocity such as GaAs this has necessitated surface passivation^7^,^10^,^11^,^17^,^18^,^25^, which adds to the complexity of device fabrication, may be incompatible with other processing steps and can also increase cavity loss through absorption^10^. Unpassivated GaAs NWs are usually characterized by a very low radiative efficiency and therefore considered unsuitable for optical applications^27^,^28^.

Here we tackle radiative efficiency by, instead, reducing the radiative lifetime of our material. Previous reports have achieved reductions in radiative lifetime through the Purcell effect by coupling emitters to a resonant cavity^29^,^30^,^31^,^32^. In contrast to these works, we employ impurity doping, which acts to directly increase radiative efficiency without the need for further fabrication steps. The resultant GaAs NWs combine excellent radiative efficiency with an ultrashort lifetime and deliver superior room temperature lasing performance relative to undoped, surface passivated GaAs/AlGaAs heterostructure NWs.

## Results

### Structural and optical characterization

**Figure 1:**
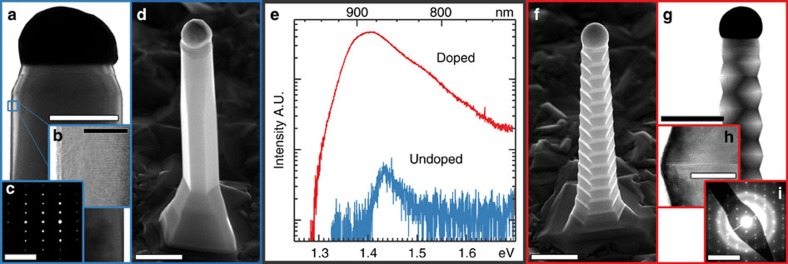
Structure, morphology and photoluminescence (PL) of undoped and doped GaAs NWs. (**a**) Bright-field TEM image of an undoped GaAs NW. Scale bar, 200 nm. (**b**) HRTEM image of the same undoped GaAs NW demonstrating the absence of stacking faults. Scale bar, 20 nm. (**c**) Electron diffraction pattern of an undoped GaAs NW corresponding to a pure wurtzite structure. Scale bar, 5 nm^−1^. (**d**) SEM image of an undoped GaAs NW. Scale bar, 500 nm. (**e**) PL spectra collected from single undoped and doped GaAs NWs under identical excitation and collection conditions. Emission from the doped NW is seen to be orders of magnitude brighter. (**f**) SEM image of a doped GaAs NW. Scale bar, 500 nm. (**g**) Bright-field TEM image of a doped GaAs NW. Scale bar, 400 nm. (**h**) HRTEM TEM image of a doped GaAs NW showing a single twin plane. Scale bar, 20 nm. (**i**) Electron diffraction pattern of a doped GaAs NW corresponding to a twinned zincblende structure. Scale bar, 10 nm^−1^. All TEM images and diffraction patterns were collected from the 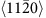/〈110〉 axis.

The GaAs NWs investigated in this work were grown by metal-organic vapour phase epitaxy at a relatively high temperature (575 °C) and low V/III ratio (1.4). In this growth regime, zinc-doping leads to a transformation from a pure wurtzite crystal structure (Fig. 1a–d) to a zincblende twining superlattice (TSL) structure (Fig. 1f-i; a high magnification transmission electron microscopy (TEM) image of the TSL structure is shown in Supplementary Fig. 1)^33^. Photoluminescence (PL) is further redshifted (1.435 to 1.402 eV) and broadened (Fig. 1e; 44 to 110 meV—see Supplementary Note 1). Most remarkably, however, we observe the intensity of PL emission to radically increase on doping. Comparing the emission of a single unpassivated and undoped GaAs NW with that of an unpassivated but optimally doped NW (Fig. 1e), the doped NW is seen to be more than two orders of magnitude brighter (∼4,700 counts) than the undoped NW (∼40 counts). This difference is even greater (450 × brighter) when integrated intensity is considered as emission from the doped NW is also spectrally broader. As the volumes of the two NWs are comparable (Supplementary Fig. 2), it is clear that the radiative efficiency of the doped NW is orders of magnitude greater than that of the undoped NW.

Previous studies have found doping to variously increase^34^,^35^ and decrease^36^ the intensity of PL emission from GaAs NWs. Increases in emission intensity have generally been linked to a mitigation of the carrier depletion arising from surface trapping^34^,^35^. An action of band-bending in suppressing surface recombination has also been considered where doping was observed to lengthen minority carrier lifetime^35^,^37^.

### Lifetime measurements

**Figure 2:**
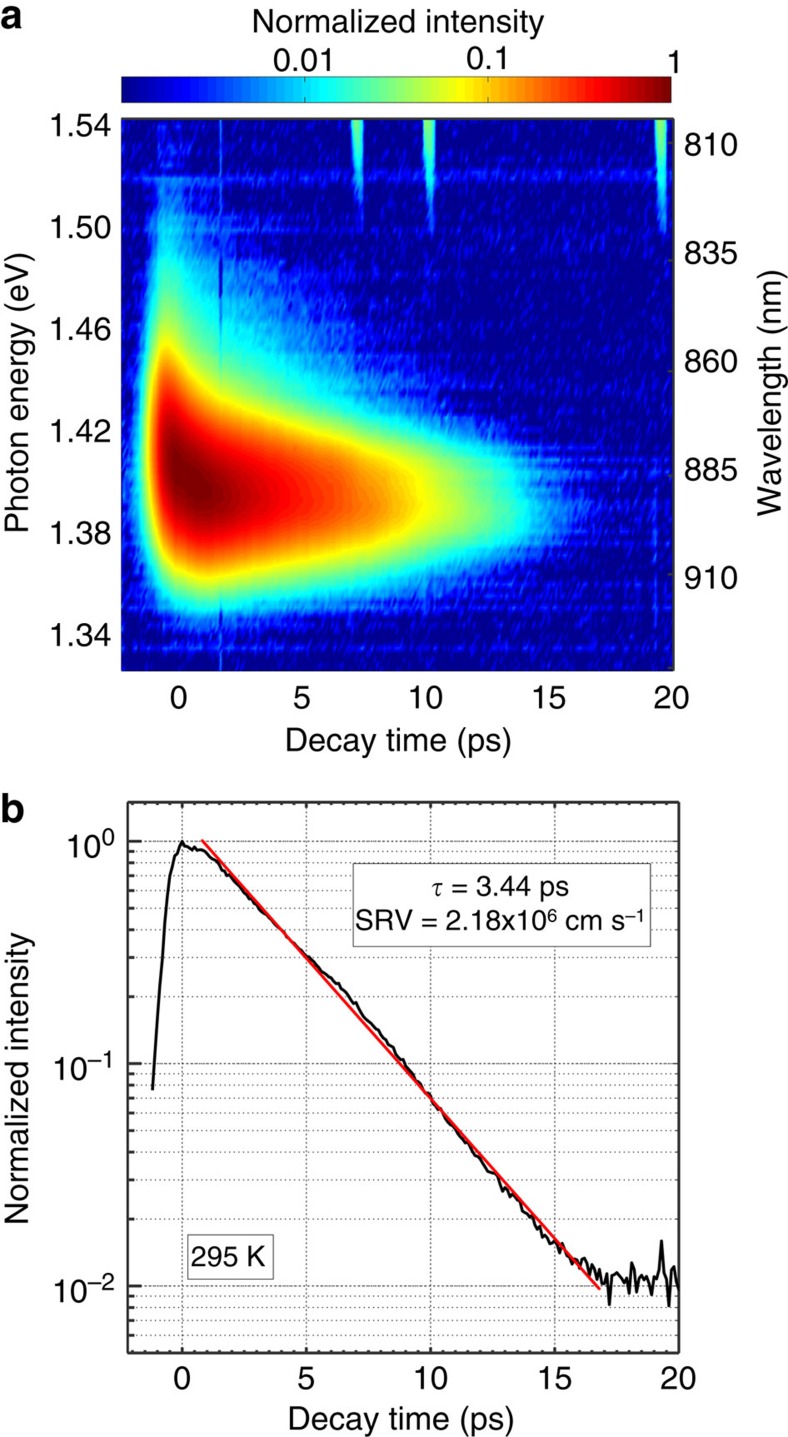
Measurement of carrier lifetime by PL up-conversion. (**a**) Emission from doped GaAs NWs resolved in energy and time. (**b**) Integration of **a** in energy, giving a lifetime of 3.44 ps and a surface recombination velocity of 2.18 × 10^6^ cm s^−1^.

Despite observing radiative efficiency to be dramatically improved by doping, we found the room temperature lifetime of our doped NWs to be only picoseconds in length (Fig. 2a,b). (The recombination dynamics were also studied by transient Rayleigh scattering spectroscopy—see Supplementary Note 2 and Supplementary Fig. 3. Low-temperature measurements are outlined in Supplementary Note 3 and Supplementary Fig. 4). This value accords well with previous studies of unpassivated GaAs NWs^35^,^37^,^38^,^39^ and represents one of the shortest carrier lifetimes reported for a semiconductor NW. Given the high surface-to-volume ratio of the NW geometry and the high rates of surface recombination known to characterize bare GaAs surfaces, the ultrashort lifetimes of bare GaAs NWs have usually been associated with non-radiative surface recombination^35^,^37^,^38^,^39^. In a regime where surface recombination dominates the dynamics of recombination, the surface recombination velocity (SRV) of a NW may be related to the minority carrier lifetime *τ*_mc_ in the following manner:


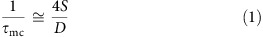


where *S* is the SRV and *D* is the NW diameter^38^,^39^,^40^,^41^. Substituting our NW diameter of 300 nm into equation (1) gives a SRV of 2.18 × 10^6^ cm s^−1^. This value is similar to that calculated previously from measurements of minority carrier diffusion length in unpassivated Zn doped GaAs NWs^35^ and towards the higher end of the range expected for bare GaAs surfaces^38^,^42^,^43^.

### Absolute efficiency measurements

**Figure 3:**
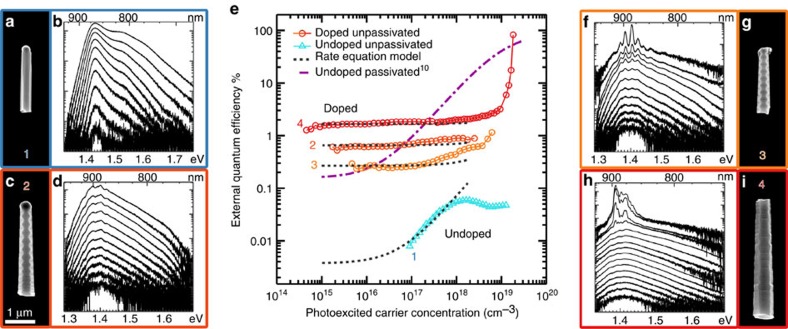
Absolute quantum efficiency measurements. (**a**) SEM image of an undoped GaAs NW and (**b**) corresponding power-dependent PL spectra. (**c**) SEM image of a doped TSL GaAs NW and (**d**) corresponding power-dependent PL spectra. (**e**) External quantum efficiency of the NWs shown in **a**,**c**,**g** and **i**. Fits of rate equation modelling are also plotted along with the modelled performance of undoped AlGaAs passivated GaAs NWs previously shown to lase at room temperature in ref. 10. (**f**) Power-dependent PL spectra of a doped TSL GaAs NW and (**g**) a corresponding SEM image. (**h**) Power-dependent PL spectra of a doped aperiodically twinned GaAs NW and (**i**) a corresponding SEM image. All SEM images are shown at the same magnification. Scale bar, 1 μm.

To further investigate the observed increase in radiative efficiency with doping, we measured the external quantum efficiency (EQE) of individual NWs as a function of pump power by calibrating our system with a known power source (see Supplementary Fig. 6 for a description of the optical setup. Nanowire absorption was modelled using finite-difference time-domain (FDTD) simulations taking into account the spot size—see Supplementary Fig. 7 and Supplementary Note 4). Figure 3 plots the EQE of four NWs of differing morphology and crystal structure along with corresponding SEM images and selected PL spectra. (The SEM images are presented without cropping in Supplementary Fig. 5) Considering first an unpassivated, undoped wurtzite GaAs NW (NW 1 in Fig. 3a), a photoexcited carrier density of at least 1 × 10^17^ cm^−3^ was required to detect PL emission (Fig. 1e). At this excitation density the measured EQE is 0.008% rising to 0.05% for carrier densities of 1 × 10^18^ cm^−3^ (light blue triangles). Saturation of the EQE at carrier densities beyond 1 × 10^18^ cm^−3^ is likely a result of several factors including photoinduced heating and extensive band-filling (see Supplementary Note 5 for a further description of PL emission from wurtzite GaAs NWs).

For the optimally doped NWs, the photoexcited carrier density required to observe PL was reduced by some two orders of magnitude to 1 × 10^15^ cm^−3^. At this carrier density, the EQE of the first doped NW (NW 2 in Fig. 3), is 0.65% and, in contrast to the undoped NW, remains approximately constant with increasing pump power. At the photoexcited carrier concentration where PL is first observed from the undoped NW (1 × 10^17^ cm^−3^) the EQE of the doped NW is two orders of magnitude greater than that of the undoped NW. Until this carrier density, the EQE of the doped NW is also greater than that of AlGaAs passivated GaAs NWs previously shown to lase (purple dash-dotted line)^10^.

Similarly enhanced values of EQE exhibiting a limited power dependence were found for all the doped NWs investigated. Figure 3 presents two further doped NWs grown under the same reactor conditions as NW 2 (Fig. 3c). The first is a TSL structure (NW 3 in Fig. 3g) grown at a standard areal density while the second is a highly defective zincblende structure (NW 4 in Fig. 3i) grown at a lower areal density (Supplementary Note 6; Supplementary Figs 8,9 and 10). The EQE of both NWs is seen to be around 1% (Fig. 3e), remaining relatively constant with pump power until the highest of excitation intensities. That the power-dependant EQE of these two very morphological different NWs is comparable, confirms that the changes in crystal phase and morphology with doping are not responsible for the observed increase in internal quantum efficiency (IQE).

The mechanism by which doping acts to increase IQE may be understood by recalling that, as a bimolecular process, the absolute rate of radiative recombination 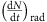 is an increasing function of the majority carrier concentration. Specifically, for a p-type material:


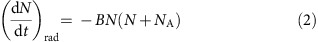


where *N* is the photoexcited carrier density, *N*_A_ is the ionized acceptor density and *B* is the radiative recombination coefficient. Now, where doping is significant, [*N*_A_≈(*N*+*N*_A_)] the radiative lifetime found by solving equation (2) will be inversely proportional to *N*_A_ (for *N*_A_≫*N*: *τ*_r_≃1/*BN*_A_). The radiative lifetime is, however, further related to IQE (*η*_IQE_) in the following manner:


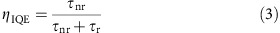


where *τ*_nr_ is the non-radiative lifetime. Inspecting equation (3), it may be appreciated that where *τ*_r_≫*τ*_nr_, as is the case for our NWs and many nanomaterials due to their large surface to volume ratio, *τ*_r_ will also be inversely related to IQE (for *τ*_r_≫*τ*_nr_: *τ*_r_≃*τ*_nr_/*η*_IQE_). Combining these statements, it is clear that where the photoexcited carrier density is lower than the ionized impurity density and the non-radiative lifetime is shorter than the radiative lifetime, IQE will be directly proportional to *N*_A_ (for *N*_A_≫*N* and *τ*_r_≫*τ*_nr_: *η*_IQE_ ∝ *N*_A_).

Returning to equation (2), we note that where *N*≫*N*_A_, the rate of radiative recombination will increase as *N*^2^ and IQE will increase with pump power as is observed for the undoped NWs. Where the reverse is however true and *N*_A_≫*N*, the rate of radiative recombination will increase as *N* and IQE will be constant with pump power as is observed for the doped NWs.

While the above rate equation analysis provides a good description of our experimental results, it does not take into account the effects of surface Fermi-level pinning^27^,^35^. To explore the potential contribution of these effects we undertook drift-diffusion type modelling (Supplementary Note 7; Supplementary Figs 11–14; Supplementary Table 1). At relatively low photoexcitation intensities, surface Fermi-level pinning was found to generate significant carrier depletion in undoped NWs reducing their IQE relative to heavily doped NWs by up to 11 orders of magnitude. For the experimental regimes investigated here, however, the relatively high doping densities or photoexcitation intensities employed were found to effectively screen this surface depletion. Similar behaviour has been reported by previous authors^37^,^44^,^45^.

As the effects of surface Fermi-level pinning may thus be considered negligible under our experimental conditions of pulsed excitation, we used a simple rate equation to fit our efficiency data (dashed black lines Fig. 3e; Supplementary Note 8; Supplementary Figs 15,16 and 17). Fitting only the rising portion of the data set collected from the undoped NW, we obtain a SRV of 2.0 × 10^6^ cm s^−1^. This velocity is very similar to that determined earlier for the doped NWs and suggests that p-type doping with zinc does not significantly alter SRV. Taking the SRV found by PL up-conversion and fitting the remaining EQE data sets, we obtain ionized dopant concentrations of 8.4 × 10^18^, 3.6 × 10^18^ and 2.5 × 10^19^ cm^−3^ for the doped NWs shown in Fig. 3c,g,i, respectively. Previous NW studies have found dopant incorporation to be higher in the radial rather than axial direction^46^,^47^ and it is therefore interesting to note that the dopant concentrations found here scale with NW diameter (the relationship between NW diameter and EQE is outlined in Supplementary Note 9 and Supplementary Fig. 18).

Returning to the power-dependent spectra presented for each of the doped NWs (Fig. 3d,f,h), regular modulations are noted in all spectra. In the case of the last two doped NWs (Fig. 3f,h), the modulations become increasingly prominent at higher pump intensities. Taken together, these observations are suggestive of amplified spontaneous emission (ASE) arising from optical feedback within the NW cavity^3^,^5^,^11^. Unlike previous work, however^10^,^16^, ASE is observed here from relatively low pump intensities. This is because the threshold for ASE scales directly with the radiative lifetime of a material^48^, which in our case has been reduced by several orders of magnitude by doping.

### Characterization of lasing in unpassivated nanowires

**Figure 4:**
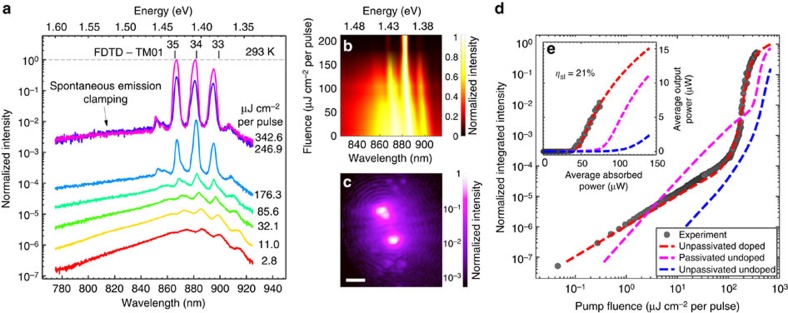
Room temperature characterization of an unpassivated GaAs NW nanolaser. (**a**) Power-dependant spectra. Note the appearance of ASE from the lowest of pump intensities and the clamping of spontaneous emission at higher pump intensities. The modelled position of several TM01 cavity modes closely match the experimental data. (**b**) Normalized spectral map across pump intensities close to threshold. (**c**) Logarithmic map of a composite optical image showing the lasing NW above threshold. (**d**) Logarithmic plot of integrated intensity of emission as a function of pump power. The clear non-linearity here represents a transition to lasing. Also shown is a fit to the experimental data (dashed red line) and modelling for undoped NWs (dashed blue and pink lines for unpassivated and passivated, respectively). Normalization here is by the highest intensity of the modelled fit. (**e**) Linear scale plot of **d** revealing slope efficiency, *η*_sl_.

Around the same photoexcited carrier concentration (∼1.0 × 10^19^ cm^−3^) that ASE becomes more prominent in the spectra of NW 4 (Fig. 3h) a superlinear increase in EQE is also noted (Fig. 3e). This superlinear increase marks a transition from spontaneous emission to lasing and was observed at room temperature for many of the doped NWs analysed. In Fig. 4, we investigate this transition and the subsequent laser action of one particular doped NW (an SEM image of this NW is shown in Supplementary Fig. 19a). Considering first the emission from this NW at a relatively low pump fluence (2.7 μJ cm^−2^ per pulse), a broad spectrum (full width at half-maximum (FWHM) ∼100 meV) centred around 1.407 eV (881 nm) is noted (Fig. 4a). Despite the low pump fluence, modulations corresponding to ASE (peak spacing ∼12 nm, FWHM ∼6 nm) are clearly apparent. Initial increases in excitation produce spectral blueshift (Fig. 4a,b), which we attribute to a carrier-induced change in refractive index. On a log–log plot, integrated intensity rises linearly with pump power in this low excitation regime (Fig. 4d) as is expected for spontaneous emission. Optical images collected in this regime show emission to be uniform along the length of the NW (Supplementary Fig. 19b).

From a pump fluence of ∼65 μJ cm^−2^ per pulse, the peaks corresponding to ASE are seen to experience gain, spectrally narrowing and increasing in intensity more rapidly than the broad spontaneous emission background. This process becomes especially pronounced around a pump fluence of 165 μJ cm^−2^ per pulse as the lasing threshold is approached. Around threshold, integrated intensity is seen to exhibit a superlinear relationship to pump fluence. For pump fluences greater than ∼200 μJ cm^−2^ per pulse the broad spontaneous emission background becomes clamped and lasing is observed from three cavity modes located at 867, 881, 895 nm (FWHM ∼2.6 nm). The relationship between integrated intensity and pump power is again linear in this regime with the gradient here corresponding to a slope efficiency of 21% (Fig. 4e). An optical image of the NW lasing (Fig. 4c) shows emission to be predominantly from the ends of the NW in the manner of a Fabry–Pérot-type cavity. The observed interference fringes indicate that the emission from these two ends is spatially coherent.

To identify the cavity modes from which lasing is observed we performed FDTD simulations (see Supplementary Note 10 and Supplementary Fig. 20). The mode with the lowest threshold gain (*g*_th_) requirement was found to be the TM01 mode, with *g*_th_=1,050 cm^−1^ and Q ∼300. Plotting the spectral position of several resonant TM01 cavity modes (Fig. 4a; also determined through FDTD modelling), modes of axial order 33–35 are seen to provide a close match to the experimentally observed lasing peaks. The simulated Q factor for these modes is furthermore in good agreement with the experimentally measured FWHM.

In Fig. 4d,e, we fit the power-dependent emission behaviour of our doped NW laser with a rate equation model (dashed red line; see Supplementary Note 11, Supplementary Fig. 21 and Supplementary Table 2). A microscopic gain model was used here to describe the doped GaAs gain medium (see Supplementary Note 12, Supplementary Fig. 22 and Supplementary Table 3) while the non-radiative and radiative recombination rate constants were taken from the literature (Supplementary Note 8). The fit shown gives an ionized doping concentration of 2.0 × 10^19^ cm^−3^, a beta factor (*β*) of 0.015 and *g*_th_ of 1,300 cm^−1^. These estimates are consistent with our fits in Fig. 3, previous publications^10^,^16^ and threshold gain modelling, respectively.

For the purposes of comparison, we also modelled the power-dependent emission behaviour of undoped NWs equal in size to the lasing NW (Fig. 4d,e). In the case of the undoped and unpassivated NW (dashed blue line), poor IQE was found to preclude laser action with the threshold pump fluence being calculated to be greater than that required for thermal vaporization. Surface passivation enables lasing (dashed pink line), but despite a relative advantage in IQE, the threshold is seen to occur at a higher pump fluence than that of our doped but unpassivated NW. The reason for this is that in addition to increasing IQE, doping also acts to shift the Fermi-level to a lower energy increasing differential gain and reducing the transparency carrier density (Supplementary Note 12). A further effect of this increase in differential gain is seen in the superior slope efficiency of the doped NW (Fig. 4e). Despite being unpassivated, we thus expected our doped NW laser to outperform undoped GaAs/AlGaAs heterostructure nanolasers of similar dimensions.

## Discussion

In conclusion, we have employed controlled impurity doping to enable room temperature lasing in unpassivated GaAs NWs, a nanomaterial usually characterized by poor radiative efficiency. Doping here acts to radically increase the radiative efficiency of our NWs while also increasing differential gain and reducing the transparency carrier density. Unlike many previous attempts to improve the efficiency of semiconductor nanomaterials by reducing the rate of non-radiative recombination, the improvement here is related to an enhanced rate of radiative recombination. Due to the fundamental nature of our approach it has general applicability to semiconductor nanomaterials regardless of device geometry or operating wavelength and is complementary with more conventional strategies such as passivation. In a device context, doping is important for achieving electrical injection and an enhanced rate of radiative recombination promises an increased modulation bandwidth^49^,^50^. We thus introduce controlled impurity doping as a simple and convenient tool for improving the radiative efficiency and performance of semiconductor nanomaterials.

## Methods

### Nanostructure growth

Au-seeded GaAs NWs were grown on semi-insulating GaAs(111)B substrates via metal-organic vapour phase epitaxy utilizing a horizontal flow reactor (Aixtron 200/4) operating at a pressure of 100 mbar and a total flow of 15 standard lines per min. Before growth, the substrates were treated with poly-L-lysine (PLL) and then a 250 nm colloidal Au solution (Ted Pella, Inc.). Following an annealing step of 10 min at 600 °C under AsH_3_, growth was performed at a temperature of 575 °C under AsH_3_ and trimethylgallium molar fractions of 1.43 × 10^−5^ and 1.04 × 10^−5^, respectively, to give a V/III ratio of ∼1.4. The dopant, diethylzinc, was introduced at molar fractions of up to 1.4 × 10^−4^ after growth of an initial undoped stem segment for 2 min.

### Electron microscopy

SEM and TEM were performed utilizing a FEI Helios 600 NanoLab Dualbeam (FIB/SEM) operated at 10 kV and a JEOL 2100F TEM operated at 200 kV, respectively. Samples for TEM investigation were prepared by mechanical dispersion on holey carbon copper grids.

### Microphotoluminescence

Microphotoluminescence (μ-PL) and lasing experiments employed a frequency doubled solid-state laser (femtoTRAIN IC-Yb-2000, *λ*=522 nm, repetition rate 20.8 MHz, pulse length 400 fs, spot size ∼5 μm) with excitation and collection through a 100x/0.9 NA (numerical aperture) objective (Nikon LU Plan) at room temperature. The collected light was spectrally filtered to remove the pump wavelength and then dispersed through a 150 lines per mm grating of a 0.75-m spectrometer. For the PL and lasing experiments the NWs were transferred onto a SiO_2_ substrate. For measurements of absolute quantum efficiency a laser diode (TT Electronics OPV302—850 nm) was used to calibrate the system. NW absorption and emission was modelled using a commercial FDTD software package (Lumerical FDTD solutions, www.lumerical.com)

### Time-resolved photoluminescence

Time-resolved PL was acquired using PL up-conversion with 30-fs time resolution. The sample was excited by 100-μJ pulses from a non-collinear OPA (NOPA) (Light conversion –Orpheus-N) pumped by a Y:KGW amplified laser system (Light Conversion—Pharos). The repetition rate was 125 kHz and central wavelength 740 nm. The emission from the NW samples was mixed with the gate pulse (from the same source) in a 50-μm-thick type-II BBO crystal. The mixing signal (centred at 402 nm) was spectrally resolved and detected by a CCD. The delay of the gate pulse was scanned in 20-fs steps.

### Data availability

The data that support the findings of this study are available from the corresponding author on request.

## Additional information

**How to cite this article:** Burgess, T. *et al.* Doping-enhanced radiative efficiency enables lasing in unpassivated GaAs nanowires. *Nat. Commun.* 7:11927 doi: 10.1038/ncomms11927 (2016).

## Supplementary Material

Supplementary Information Supplementary Figures 1-22, Supplementary Tables 1-3, Supplementary Notes 1-12 and Supplementary References.
