# Supplementary material for: Doping-enhanced radiative efficiency enables lasing in unpassivated GaAs nanowires
Source: Nat Commun. 2016 Jun 17;7:11927. doi: 10.1038/ncomms11927 (PMC4915017; doi:10.1038/ncomms11927)
Supplement: Supplementary Information — Supplementary Figures 1-22, Supplementary Tables 1-3, Supplementary Notes 1-12 and Supplementary References. [file ncomms11927-s1.pdf]

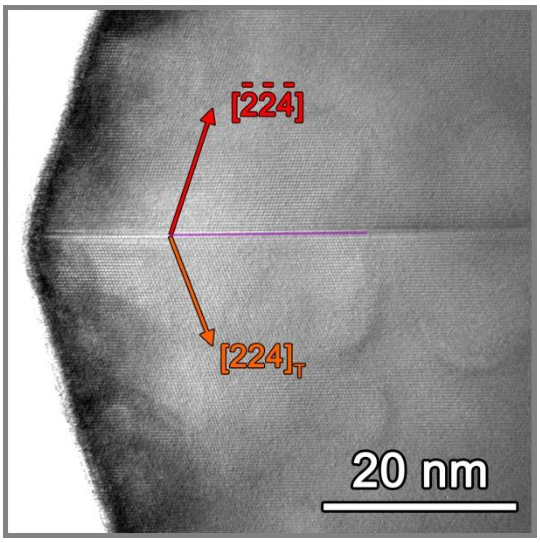


**Supplementary Figure 1 | <110> axis high-resolution TEM image of a twin boundary in a TSL NW.** In all cases examined, the transition between twins in twinning superlattice (TSL) structures was found to be abrupt and without the addition of a wurtzite segment.

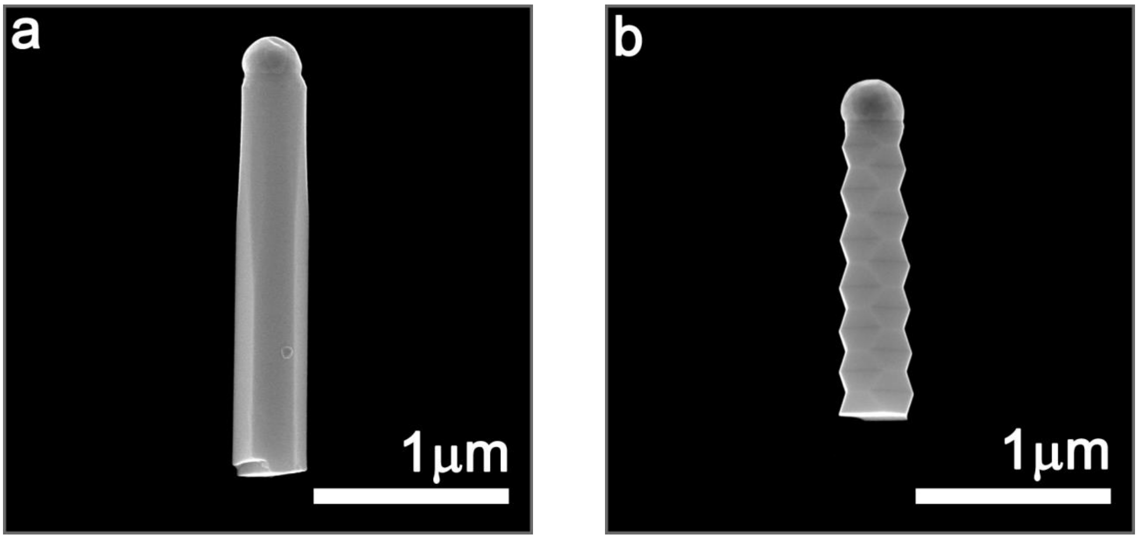


**Supplementary Figure 2 | SEM images of the NWs from which the spectra presented in Fig. 1e of the main manuscript were collected. (a)** undoped wurtzite **(b)** doped twinning superlattice type zincblende. As can be seen, both NWs have a similar tip diameter although the undoped NW in (a) is somewhat longer than the doped NW in (b). Approximating their geometry as truncated cones, the undoped NW has a tip diameter of 340 nm, a base diameter of 390 nm and a length of 2.06 μm to give a volume of 0.22 $\mu m^3$. The doped NW has a tip diameter of 299 nm, a base diameter of 350 nm and a length of 1.52 μm to give a volume of 0.13 $\mu m^3$, which is some 40% less than that of the undoped NW.

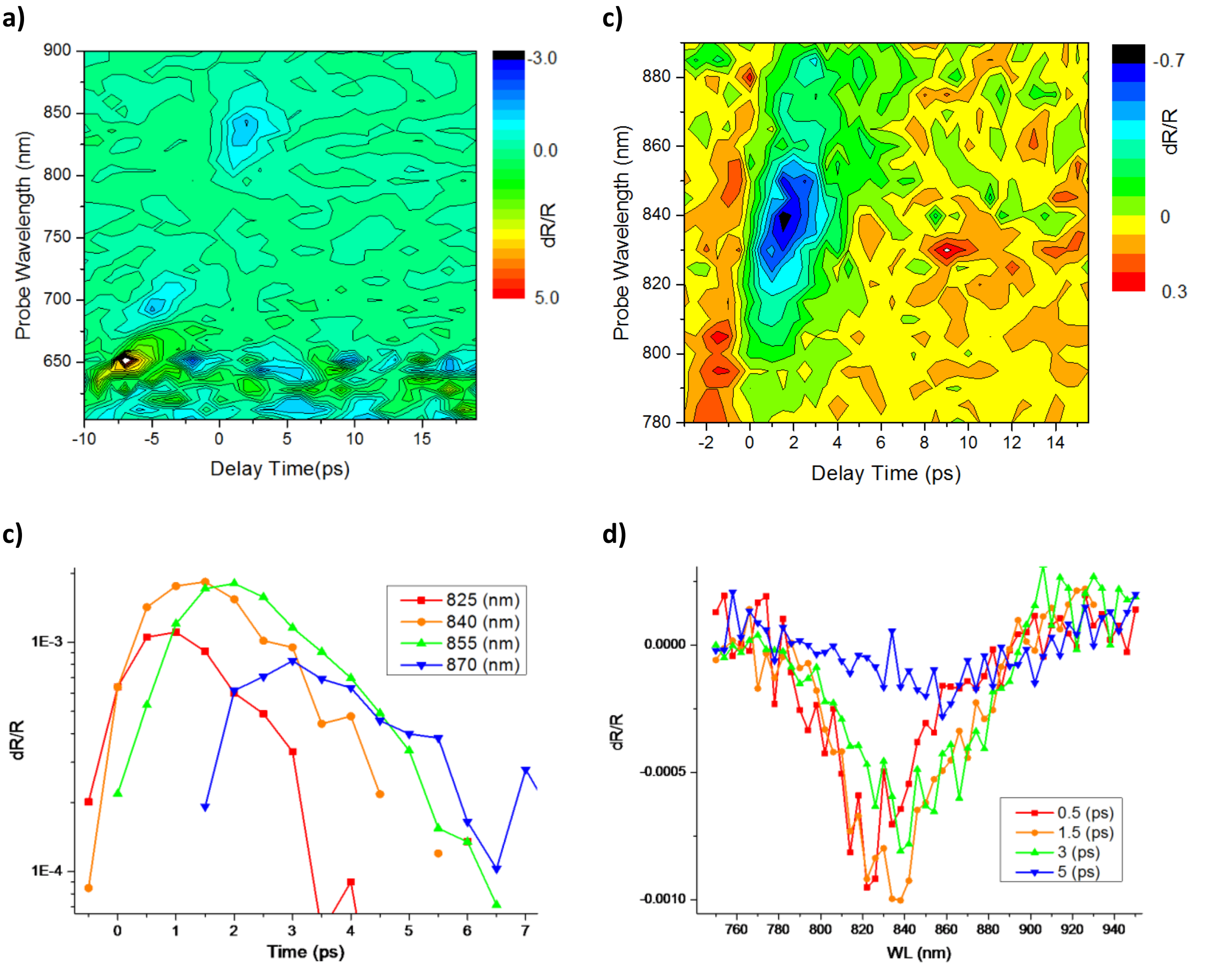


**Supplementary Figure 3 | Transient Rayleigh scattering characterisation of a single doped GaAs NW. (a)** photomodulated polarisation response ΔR'/R' **(b)** a magnified view of (a) close to the band gap **(c)** time dependant photomodulated polarisation response ΔR'/R' for several wavelengths close to the band gap **(d)** wavelength dependant photomodulated polarisation response ΔR'/R' for several times following excitation. The minority carrier lifetime is observed to be picoseconds in length in agreement with Fig. 2 of the main manuscript.

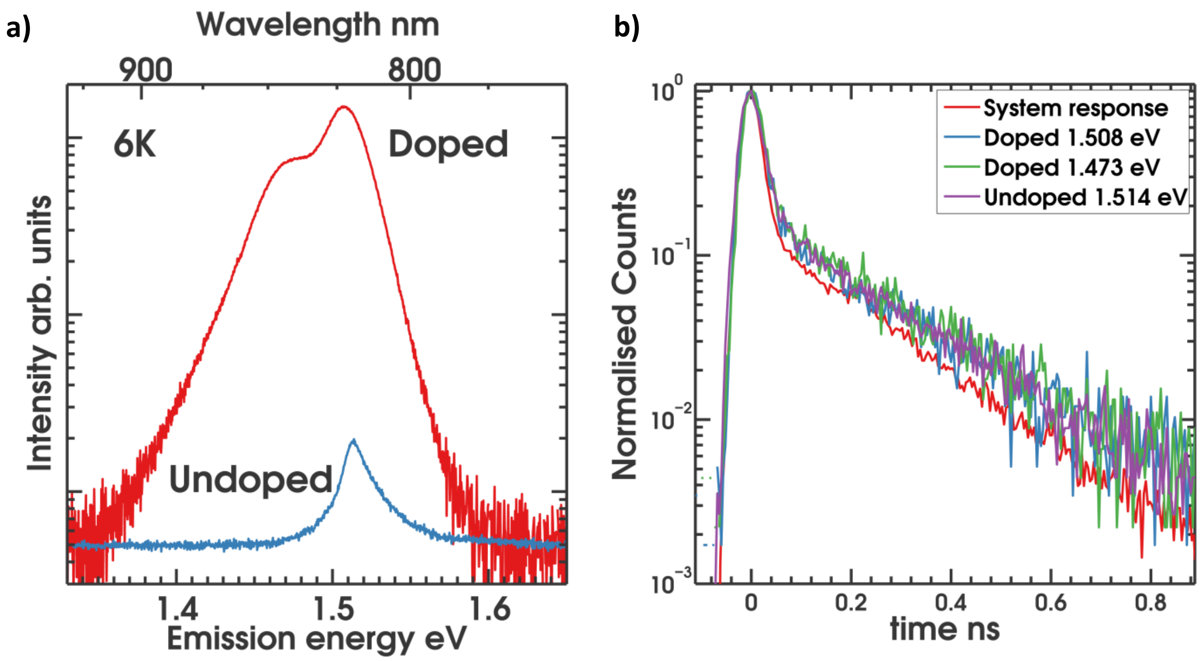


**Supplementary Figure 4 | Low temperature (6 K) PL characterisation of single NWs. (a)** Emission spectra normalized to an excitation fluence of 5 μJ cm$^{-2}$ per pulse **(b)** TCSPC measurements at the energies stated. Data is limited by the system response indicating lifetimes of less than 80 ps. The efficiency advantage obtained through doping continues at low temperature. Emission from the doped NW is again seen to be orders of magnitude brighter than that from the undoped NW.

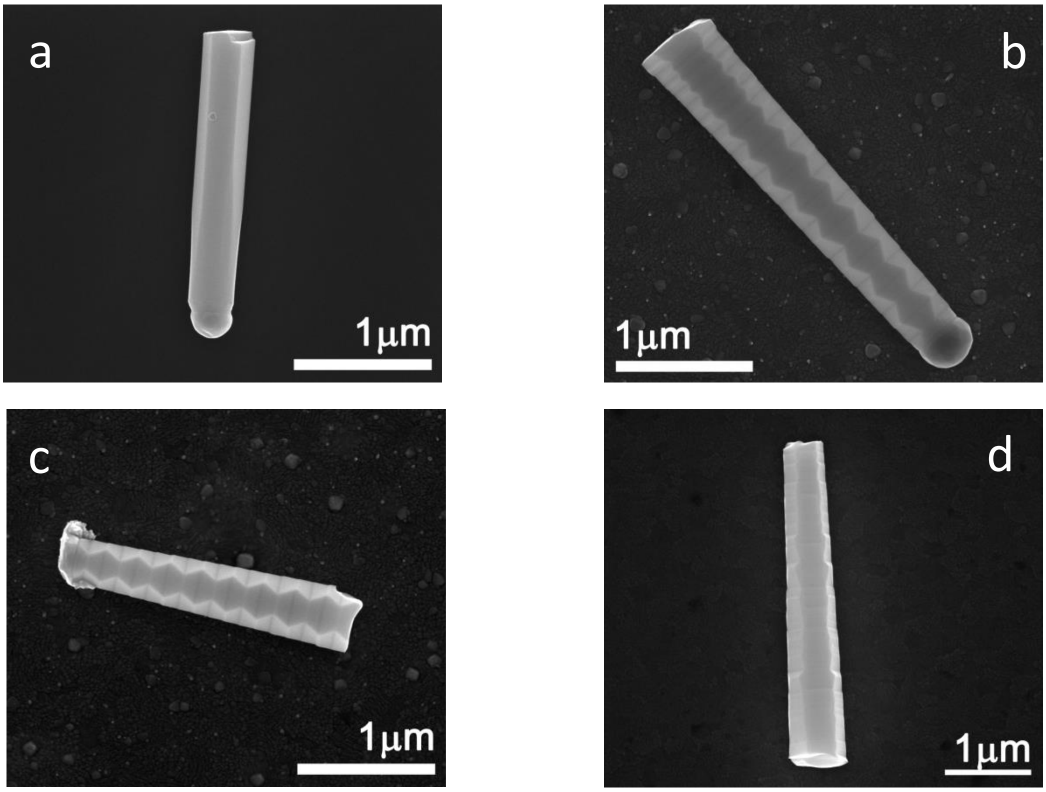


**Supplementary Figure 5 | SEM images before cropping and thresholding. (a-d)** correspond to Fig. 3(a,c,g,i) in the main manuscript respectively.

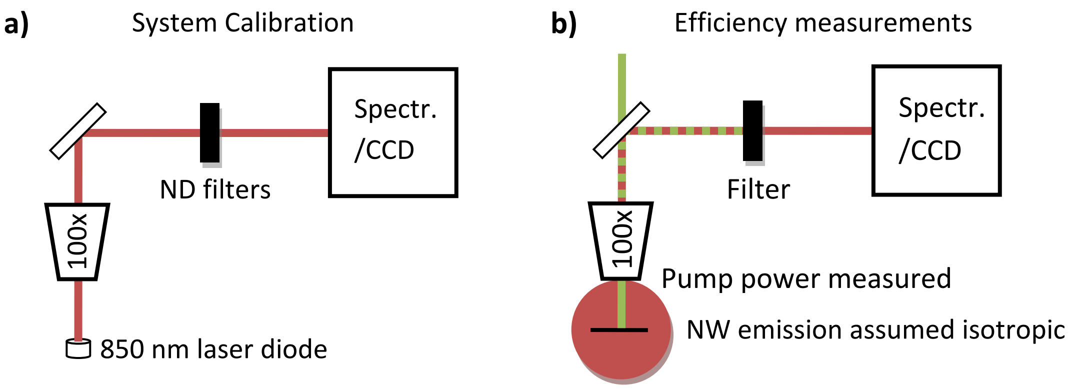


**Supplementary Figure 6 | Schematics showing the experimental setup. (a)** calibration of our CCD sensor sensitivity and **(b)** absolute external quantum efficiency measurements. A laser diode operating at 850 nm (TT Electronics OPV302) was used as the reference source. The output of this reference laser was firstly measured by an optical power meter (Thor PM100D) before its spectra was collected by our system using the same parameters as later power-dependant photoluminescence experiments. Being of small spatial size and low divergence, the entire reference beam was collected by our system enabling the ratio between photons collected and CCD counts to be measured. When calculating the absolute external efficiency of our NWs, we assumed light emission to be isotropic.

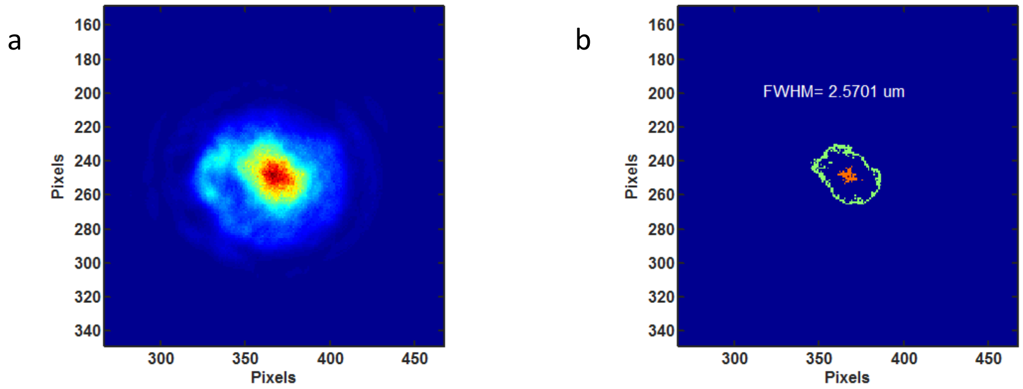


**Supplementary Figure 7 | Measurement of excitation spot size. (a)** An image of the reflected laser spot **(b)** Analysis of (a) showing the brightest 50 pixels in orange and pixels with 45-55% brightness in green. The FWHM was calculated as the mean distance between the green pixels and the centroid of the orange pixels. Distance was calibrated from corresponding SEM measurements and by using a calibration slide.

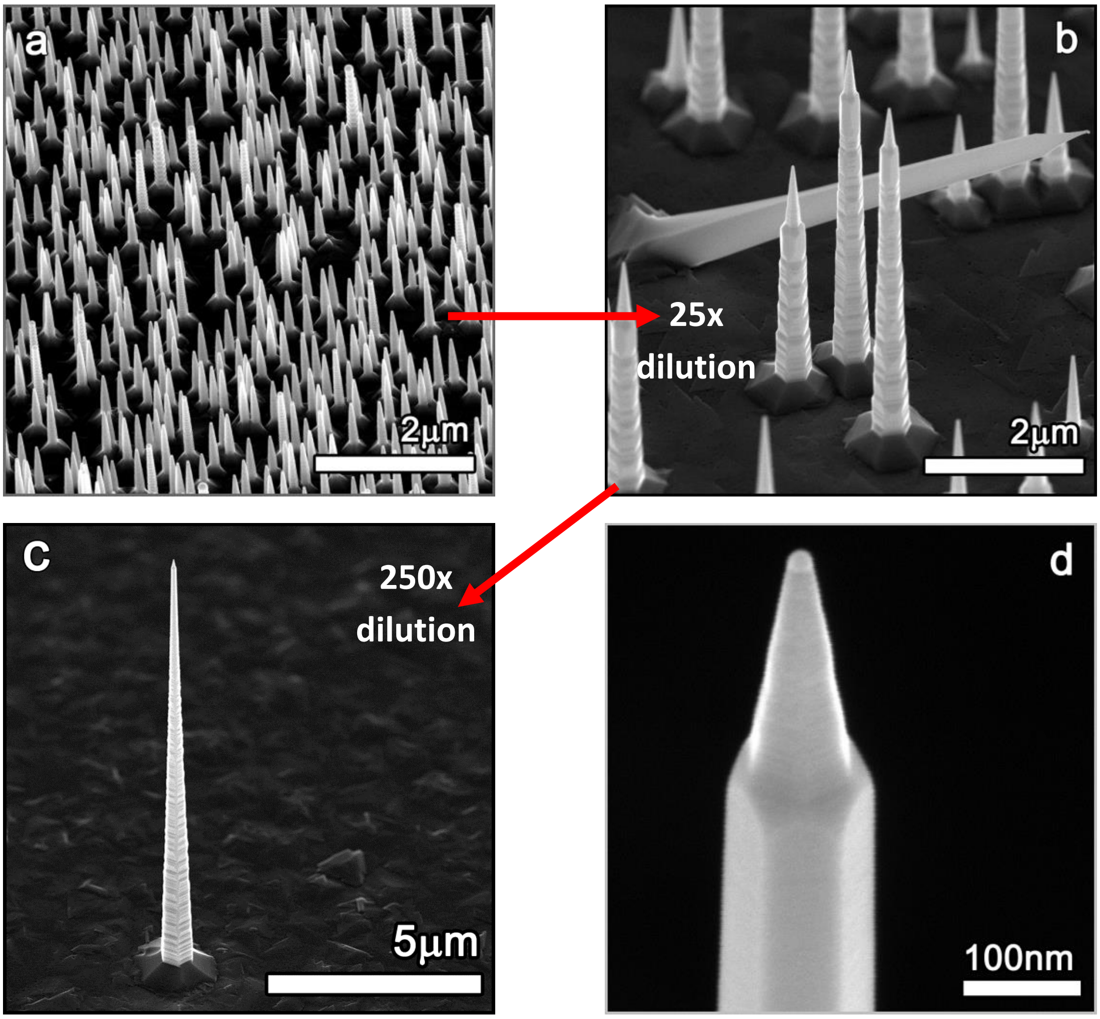


**Supplementary Figure 8 | SEM images showing the morphology of doped NWs grown at different areal densities. (a)** Normal density **(b)** 25x dilution **(c)** 250x dilution **(d)** magnification of the tip of the NW shown in (c).

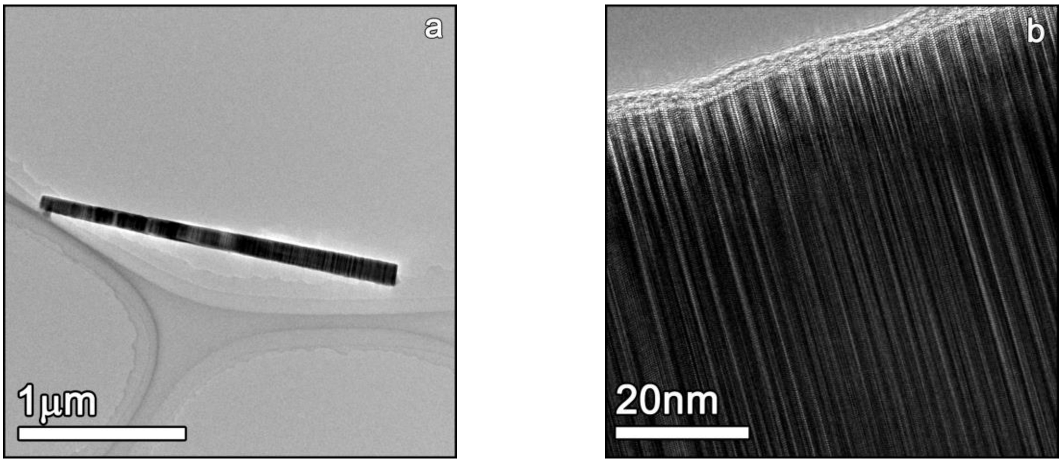


**Supplementary Figure 9 | <110> axis TEM images of a GaAs NW grown at low areal density revealing a mixed phase structure. (a)** Low-magnification image showing tapering **(b)** High-magnification image showing a mixed phase structure with a high density of planar defects.

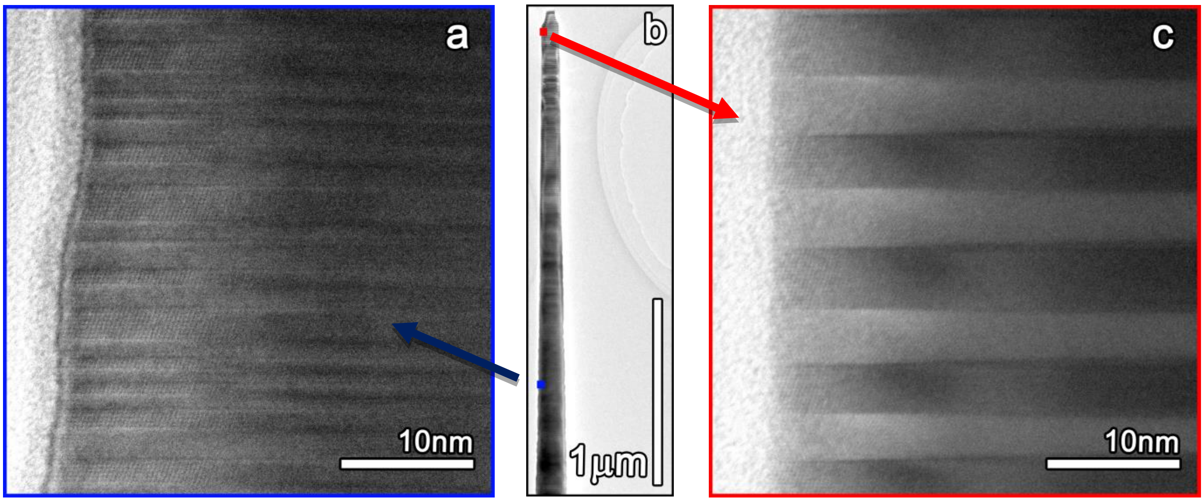


**Supplementary Figure 10 | <110> axis TEM images of a GaAs NW grown at low areal density showing a transition from mixed phase to TSL structure. (a)** High-magnification image collected near to the base of the NW revealing a mixed phase structure. **(b)** Low-magnification image of the NW **(c)** High-magnification image collected near to the tip of the NW revealing a periodic TSL structure exhibiting {110} type sidewalls.

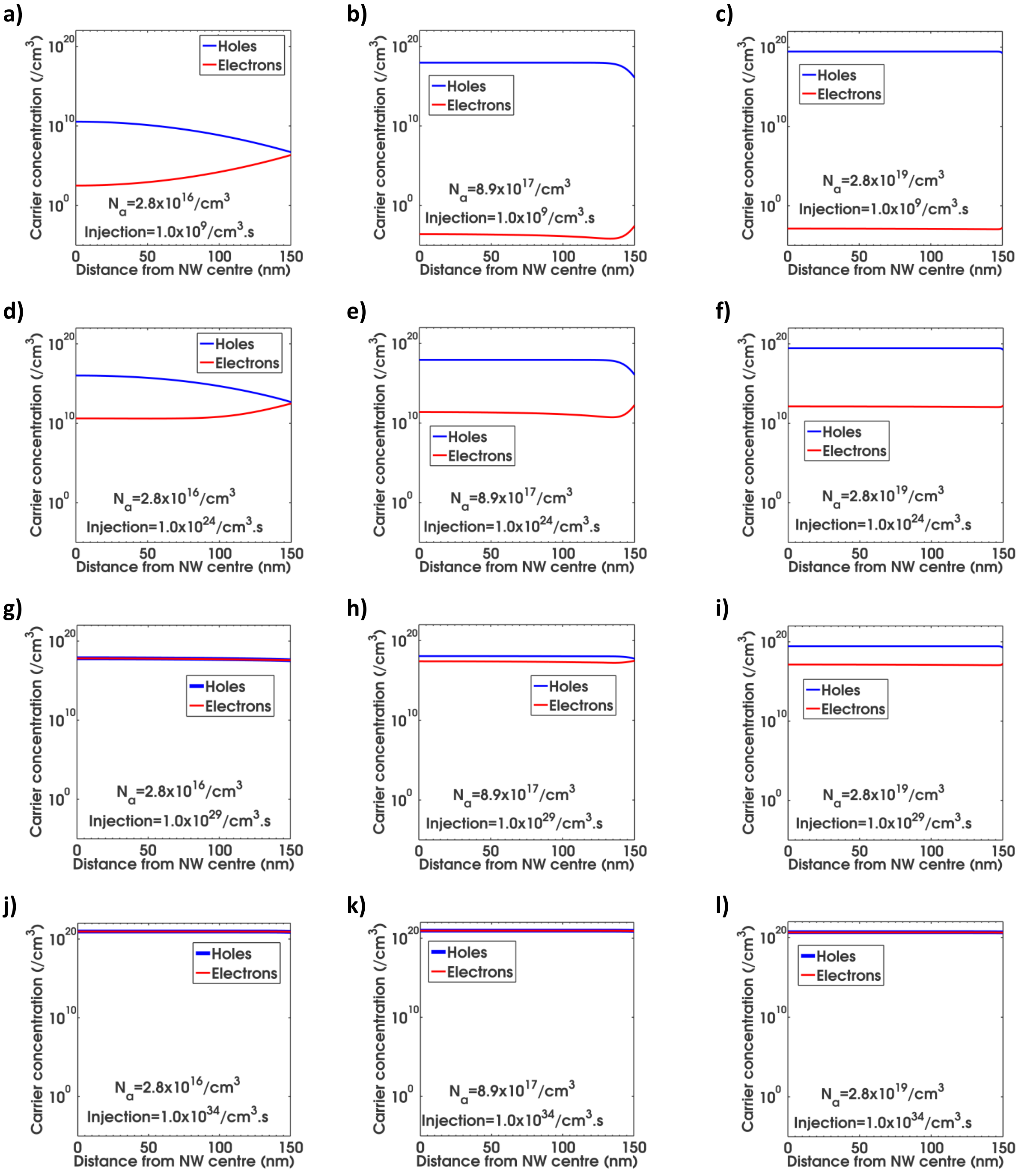


**Supplementary Figure 11 | Modelled carrier concentration along the radial direction for various doping densities and carrier generation rates. (a)** $N_A$= 2.8x10$^{16}$ cm$^{-3}$, Injection= 1.0x10$^{9}$ cm$^{-3}$ s$^{-1}$ **(b)** $N_A$ = 8.9x10$^{17}$ cm$^{-3}$, Injection= 1.0x10$^{9}$ cm$^{-3}$ s$^{-1}$ **(c)** $N_A$ = 2.8x10$^{19}$ cm$^{-3}$, Injection= 1.0x10$^{9}$ cm$^{-3}$ s$^{-1}$ **(d)** $N_A$ = 2.8x10$^{16}$ cm$^{-3}$, Injection= 1.0x10$^{24}$ cm$^{-3}$ s$^{-1}$ **(e)** $N_A$ = 8.9x10$^{17}$ cm$^{-3}$, Injection= 1.0x10$^{24}$ cm$^{-3}$ s$^{-1}$ **(f)** $N_A$ = 2.8x10$^{19}$ cm$^{-3}$, Injection= 1.0x10$^{24}$ cm$^{-3}$ s$^{-1}$ **(g)** $N_A$ = 2.8x10$^{16}$ cm$^{-3}$, Injection= 1.0x10$^{29}$ cm$^{-3}$ s$^{-1}$ **(h)** $N_A$ = 8.9x10$^{17}$ cm$^{-3}$, Injection= 1.0x10$^{29}$ cm$^{-3}$ s$^{-1}$ **(i)** $N_A$ = 2.8x10$^{19}$ cm$^{-3}$, Injection= 1.0x10$^{29}$ cm$^{-3}$ s$^{-1}$ **(j)** $N_A$ = 2.8x10$^{16}$ cm$^{-3}$, Injection= 1.0x10$^{34}$ cm$^{-3}$ s$^{-1}$ **(k)** $N_A$ = 8.9x10$^{17}$ cm$^{-3}$, Injection= 1.0x10$^{34}$ cm$^{-3}$ s$^{-1}$ **(l)** $N_A$ = 2.8x10$^{19}$ cm$^{-3}$, Injection= 1.0x10$^{34}$ cm$^{-3}$ s$^{-1}$. Note that band bending is reduced by both increased doping and increased rates of carrier generation.

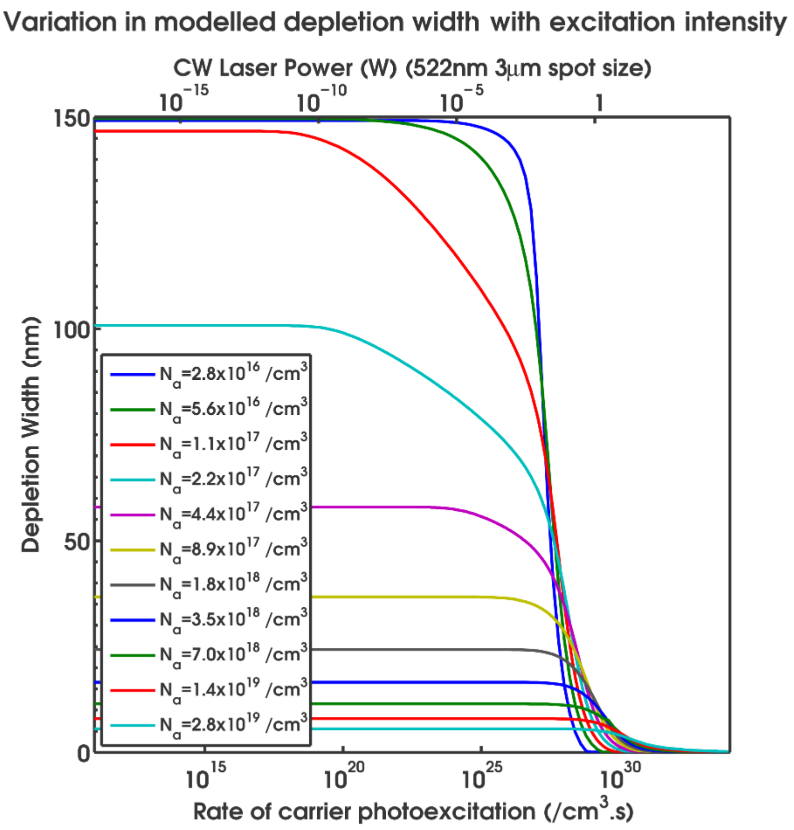


**Supplementary Figure 12 | Modelled depletion width as a function of carrier generation rate for various doping densities $N_A$ (cm$^{-3}$).**

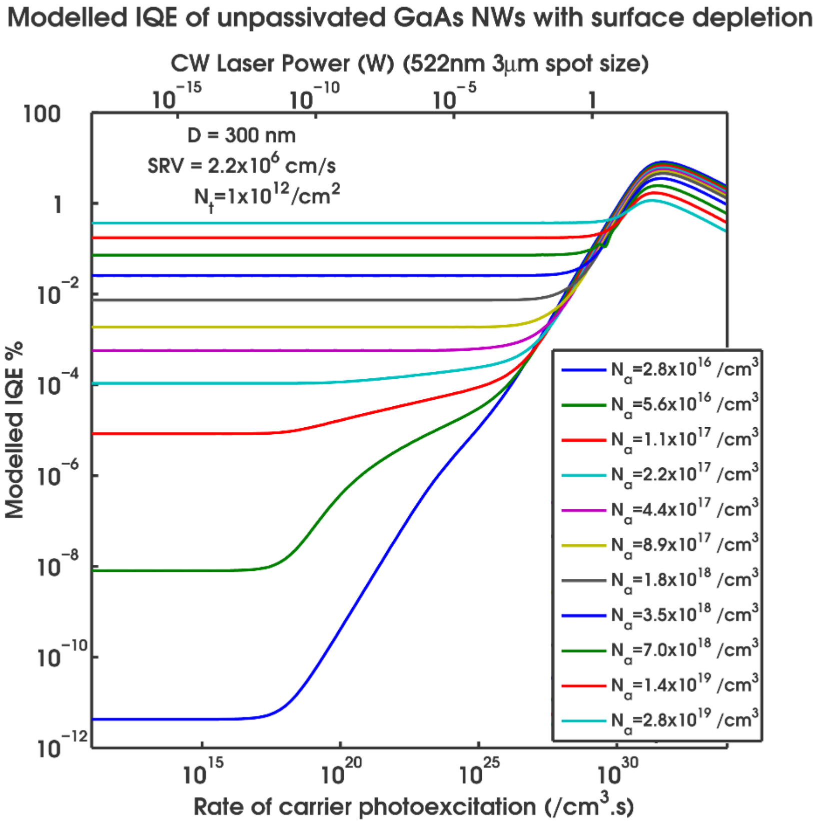


**Supplementary Figure 13 | Modelled variation of IQE with carrier generation rate for various doping concentrations ($cm^{-3}$) and a trap density $N_t$ of $1.0x10^{12}$ $cm^{-2}$.**

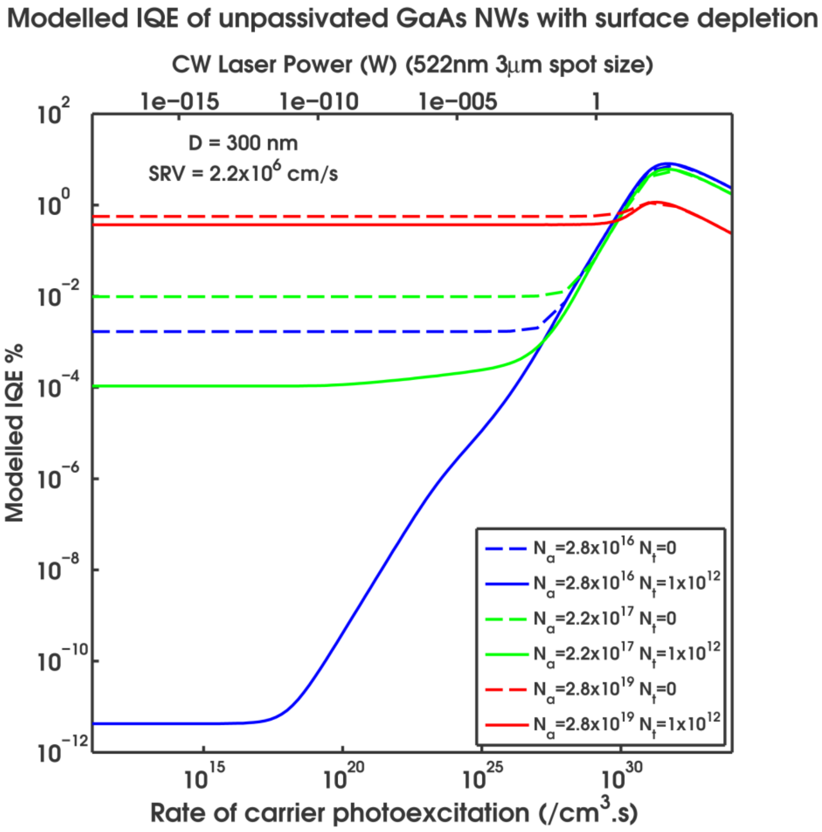


**Supplementary Figure 14 | Modelled variation of IQE with carrier generation rate with (trap density $N_t$=1x10$^{12}$ cm$^{-2}$) and without ($N_t$=0 cm$^{-2}$) surface charge trapping for various doping concentrations $N_A$ (cm$^{-3}$).**

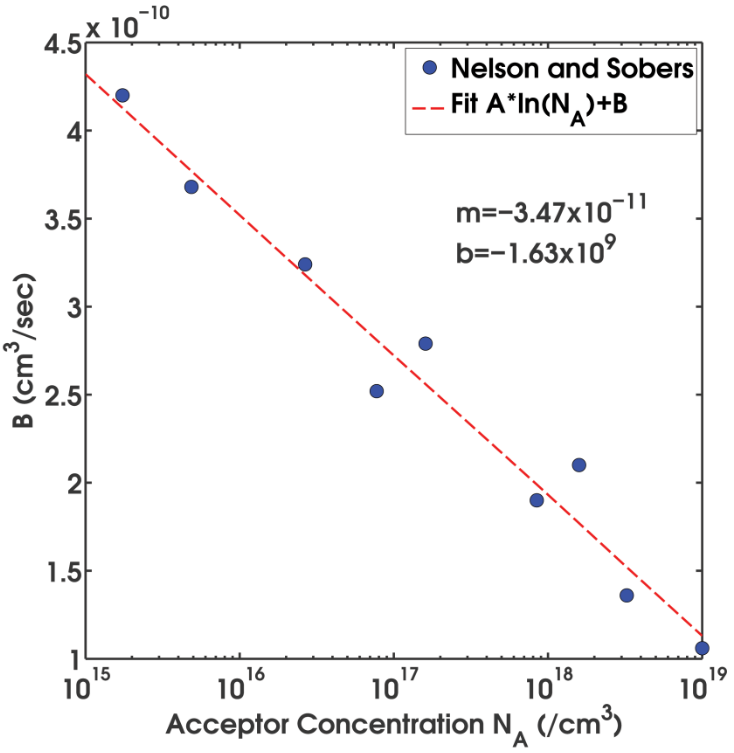


**Supplementary Figure 15 | Variation of the radiative recombination coefficient with p-type doping as determined by Nelson and Sobers[1] and the fit giving the *B* values used in this work.**

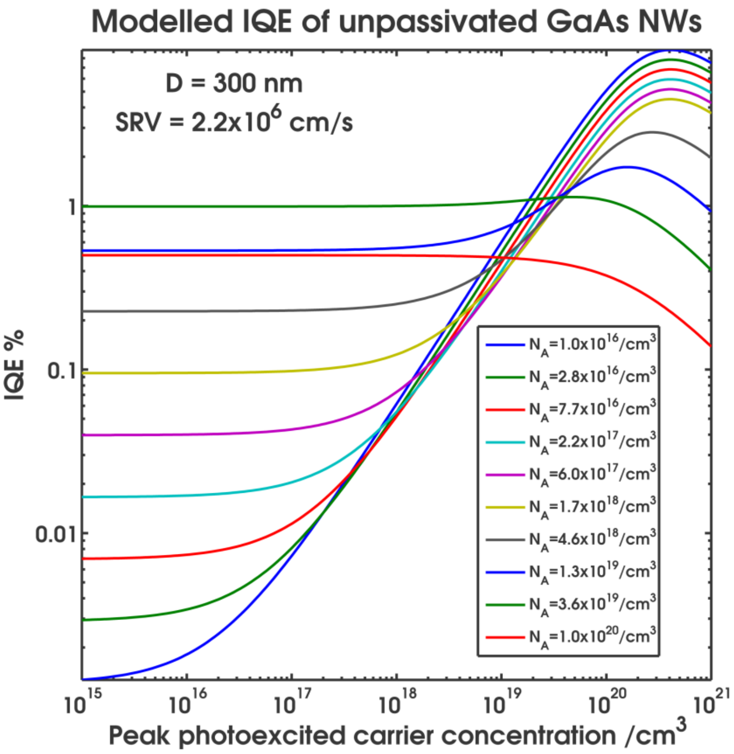


**Supplementary Figure 16 | Modelled variation of IQE with peak excitation for variously doped GaAs NWs of 300 nm diameter characterised by a surface recombination velocity of $2.2x10^6$ cm $s^{-1}$.**

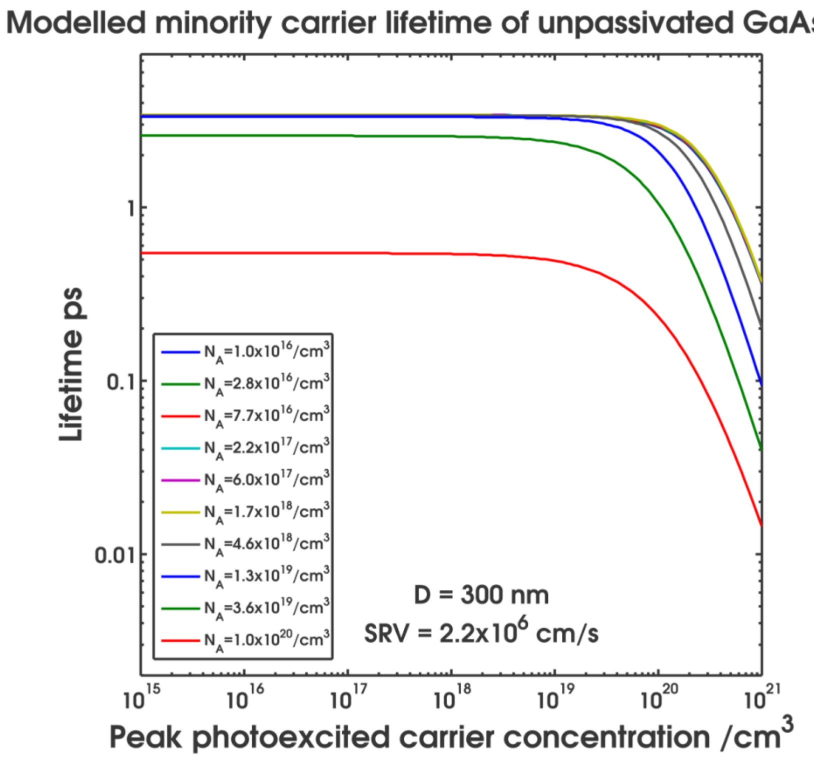


**Supplementary Figure 17 | Modelled variation of minority carrier lifetime with excitation for variously doped GaAs NWs of 300 nm diameter characterised by a surface recombination velocity of $2.2\times10^{6}$ cm $s^{-1}$.**

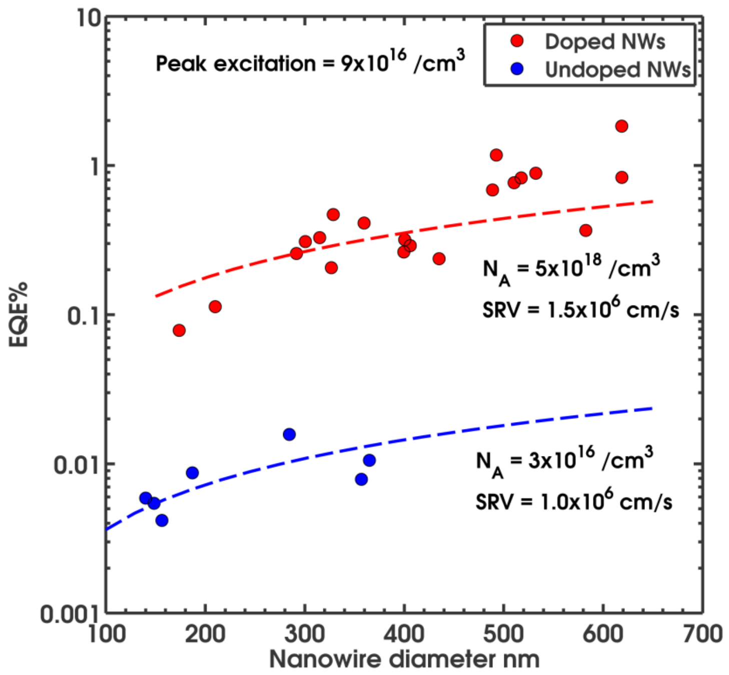


**Supplementary Figure 18 | Measured EQE as a function of diameter for doped and undoped NWs at a peak photoexcitation of $9\times10^{16}$ cm$^{-3}$.** Dashed lines present fits of Supplementary Equation 7 with the parameters shown.

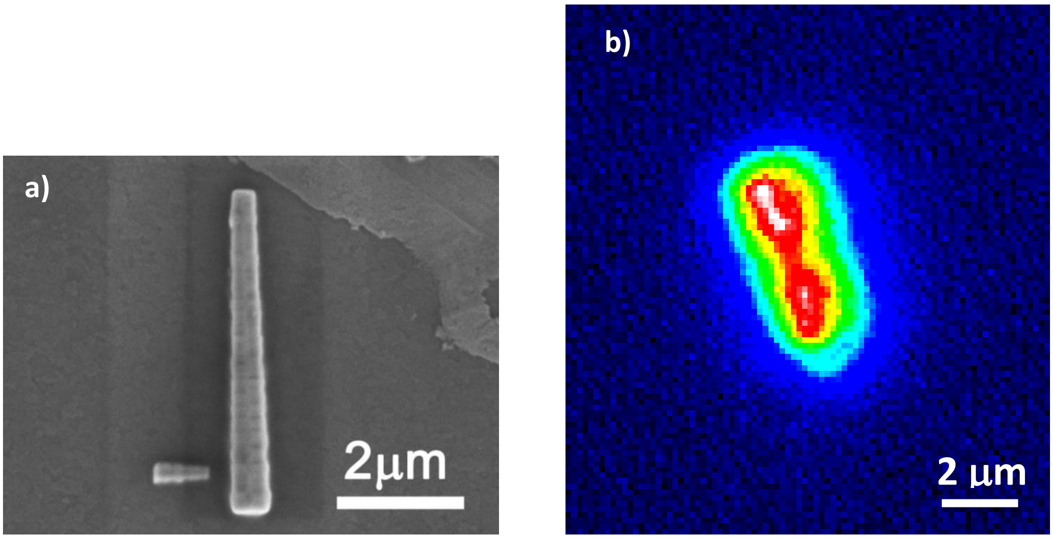


**Supplementary Figure 19 | Images of the GaAs NW from which lasing was observed as characterised in Fig. 4. (a)** SEM image. The nanowire is tapered, with the diameter varying from 340 nm at the tip to 590 nm at the base, and is 5.15 μm in length. This NW was grown at a relatively low areal density and from the appearance of the sidewalls facets has a mixed phase structure. **(b)** An optical image of emission from the NW below threshold which is seen to be relatively uniform.

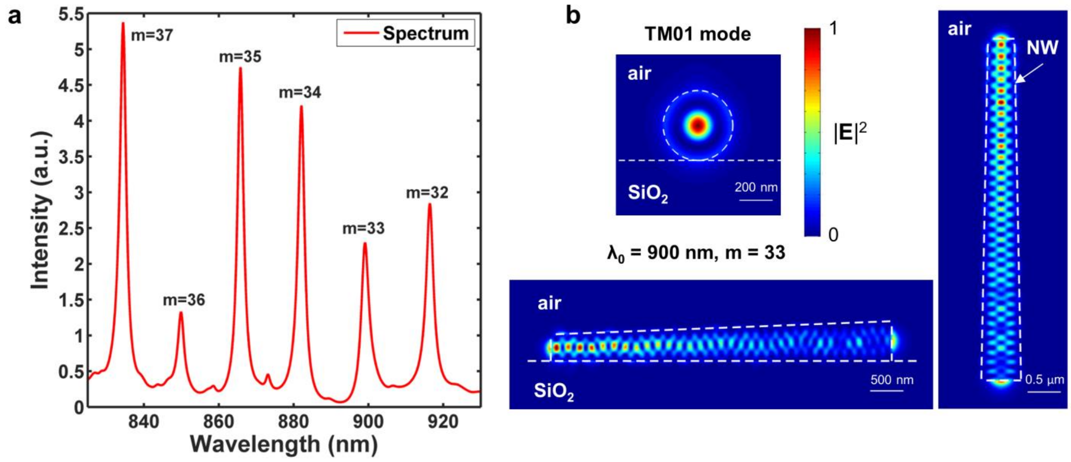


**Supplementary Figure 20 | FDTD simulations. (a)** Cavity spectrum calculated from FDTD simulation, showing the spectral position of TM01 resonant modes supported in the nanowire laser. The axial order (m) is denoted above each peak. **(b)** Electric field intensity profiles in the cross-section of the nanowire at wavelength of 900 nm. The field profiles show that the resonant mode at 900 nm corresponds to the TM01 mode with m=33.

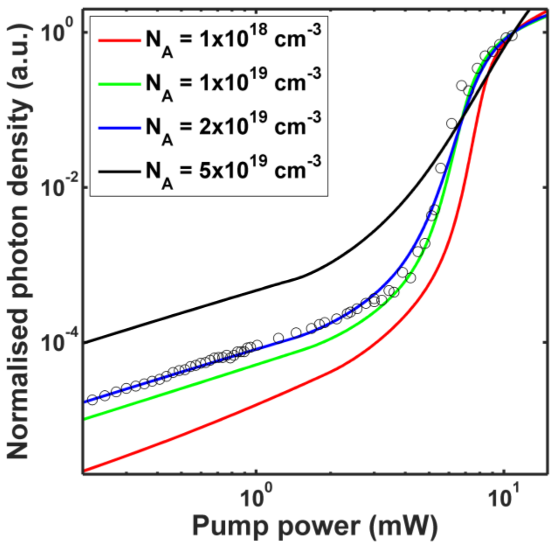


**Supplementary Figure 21 | Rate equation modelling.** L-L curves obtained from rate equation modelling, for different doping concentrations, with $g_{\mathrm{th}}$=1300 cm$^{-1}$ and $\beta$=0.015. The experimental data is superimposed (open circles).

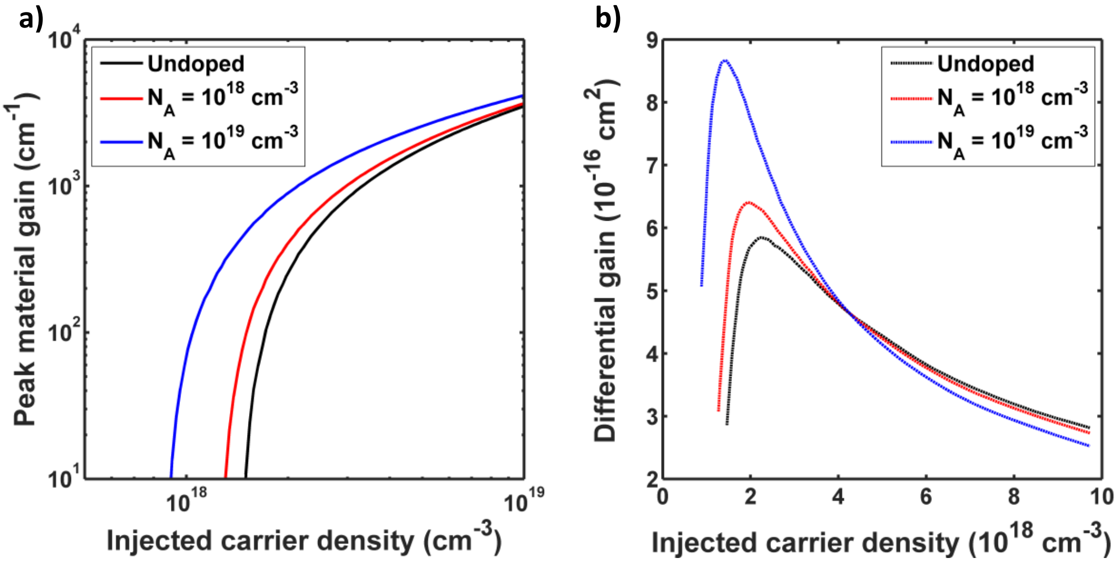


**Supplementary Figure 22 | Gain modelling. (a)** Peak material gain as function of injected carrier density for p-type doped and undoped bulk GaAs. **(b)** Differential gain as function of injected carrier density for p-type doped and undoped bulk GaAs.

**Supplementary Table 1 |** Parameters used for the finite volume modelling of carrier recombination.

| Parameter | Value |
|---|---|
| Surface trap density | $1x10^{12}$ $cm^{-2}$ |
| Surface recombination velocity | $2.2x10^{6}$ cm $s^{-1}$ |
| Electron mobility | 8500 $cm^{2}$ $V^{-1}$ $s^{-1}$ |
| Hole mobility | 400 $cm^{2}$ $V^{-1}$ $s^{-1}$ |
| Bulk SRH lifetime | 10 ns |
| Radiative recombination coefficient | Sup. Eqn. 4 |
| Auger recombination coefficient | Sup. Eqn. 5 |

**Supplementary Table 2 |** Gain function parameters for different values of $N_{\mathrm{A}}$.

| | $N_{\mathrm{A}}$= 1×10$^{18}$ cm$^{-3}$ | $N_{\mathrm{A}}$= 1×10$^{19}$ cm$^{-3}$ | $N_{\mathrm{A}}$= 2×10$^{19}$ cm$^{-3}$ | $N_{\mathrm{A}}$= 5×10$^{19}$ cm$^{-3}$ |
|---|---|---|---|---|
| $g_0$ | 950 cm$^{-1}$ | 835 cm$^{-1}$ | 780 cm$^{-1}$ | 700 cm$^{-1}$ |
| $N_{\mathrm{tr}}$ | 2.55×10$^{18}$ cm$^{-3}$ | 2.0×10$^{18}$ cm$^{-3}$ | 1.75×10$^{18}$ cm$^{-3}$ | 1.4×10$^{18}$ cm$^{-3}$ |
| $N_{\mathrm{s}}$ | -1.1×10$^{18}$ cm$^{-3}$ | -1.1×10$^{18}$ cm$^{-3}$ | -1.1×10$^{18}$ cm$^{-3}$ | -1.1×10$^{18}$ cm$^{-3}$ |

**Supplementary Table 3 |** Parameters used to model gain in doped GaAs.

| Parameter | Value |
|---|---|
| $m_{\mathrm{e}}^{*}$ | 0.067 $m_0$ kg |
| $m_{\mathrm{lh}}^{*}$ | 0.087 $m_0$ kg |
| $m_{\mathrm{hh}}^{*}$ | 0.51 $m_0$ kg |
| $\|M\|^2/m_0$ | 4.8 eV |
| $E_{\mathrm{g}}$ | $1.519 - 5.405\cdot10^{-4}\cdot T^2/(T+204)$ eV |
| $E_A$ | 24 meV |
| $n_{\mathrm{r}}$ | 3.6 |
| $\gamma$ | 6.6 meV |
| $T$ | 300 K |

## Supplementary Note 1 | Description of bandgap narrowing in doped nanowires

In comparison to undoped zincblende (ZB) GaAs nanowires,[2, 3] emission from the doped zincblende twinning superlattice (TSL) structures is redshifted and significantly broader, peaking at approximately 1.402 eV (884 nm) with a FWHM of 110 meV (see Fig. 1e main manuscript). Redshift here to energies below that expected for ZB GaAs is related to bandgap narrowing and corresponds in magnitude to a doping density of approximately $10^{19}$ $cm^{-3}$. [4-6]

## Supplementary Note 2 | Transient Rayleigh scattering

The dynamical behaviour of the doped nanowires was further explored by transient Rayleigh scattering (TRS) spectroscopy.[7] Supplementary Fig. 3a plots the photomodulated polarisation response, $\Delta R'/R' = \Delta(R_{\parallel} - R_{\perp})/(R_{\parallel} - R_{\perp})$, of a single doped TSL nanowire as a function of time and scattered wavelength. Delay time here reflects dispersion in the optics and source laser fibre with the zero time being chosen to correspond with the onset of the fundamental band gap response at around 850 nm. The spin-orbit split-off band transition is further observed at around 700 nm and appears more pronounced as $R'$ approaches zero at shorter wavelengths. By considering the response at several particular wavelengths close to the band gap (as plotted in Supplementary Fig. 3c), we estimate the minority carrier lifetime to be approximately 1 ps. This is in good agreement with the up-conversion measurement presented in Fig. 2 of the main manuscript.

Beyond carrier dynamics, TRS spectroscopy also provides insight into band structure as carriers relax towards their equilibrium distributions following excitation.[8] Supplementary Fig. 3d plots TRS spectra as a function of wavelength for several particular times following excitation. Given the minority carrier lifetime of approximately 1 ps, the response at 3 ps can be expected to represent carrier concentrations and temperatures approaching equilibrium. The minimum of this spectrum is observed at around 850 nm (1.46 eV) and corresponds to the transition between valence and conduction band edges. Blueshift here from a wavelength of 872 nm (1.42 eV) in undoped GaAs may be attributed to heavy hole doping having shifted the Fermi level to a position within the valence band. Redshift of the fundamental band gap from 872 nm (1.42 eV) to approximately 880 nm (1.41 eV), as determined from the zero crossing point (as plotted in Supplementary Fig. 3d), may further be attributed to band gap renormalisation due to doping.

Taken together we can thus approximate the quasi hole Fermi level to be +50 meV. (A full calculation taking into account temperature effects is beyond the scope of this paper.) From this energy, a heavy hole doping concentration of at least $2.4\times10^{19}$ $cm^{-3}$ and a light hole doping concentration of at least $1.5\times10^{18}$ $cm^{-3}$ may be calculated for a temperature of 300 K. Similarly the late time displacement of the split-off band to conduction band transition of +10 meV is indicative of

a doping level of at least $1.8x10^{18}$ $cm^{-3}$. These high hole doping concentrations are in agreement with our rate equation modelling of both IQE and L-L characteristic of the NW laser analysed.

### Supplementary Note 3 | Low temperature PL

In all cases the lifetime of PL emission at low temperature was found to be less than the 80 ps system response of our time correlated single photon counting (TCSPC) setup (see Supplementary Fig. 4b). Such short lifetimes are consistent with previous measurements[9-13] of GaAs nanostructures at low temperature and indicate the continued dominance of a non-radiative recombination pathway. The surface recombination velocity of GaAs has been found[12-14] to remain relatively high (>$1x10^5$ cm $s^{-1}$) at cryogenic temperatures and the lifetime of GaAs nanostructures has previously been linked to surface recombination at these temperatures.[12, 14-16] In the case of InGaAs/InP wires, surface recombination velocity was found to vary as the square root of absolute temperature between 4 and 77 K. This suggests a capture cross section that is independent of temperature. Such behaviour can be expected for traps with a large capture cross section such as those present at the surface of unpassivated GaAs.[17, 18]

### Supplementary Note 4 | Quantifying radiative efficiency

We quantified radiative efficiency by calibrating our system (see Supplementary Fig. 6), measuring spot size (see Supplementary Fig.7) and estimating NW absorption through FDTD modelling. NWs were modelled as truncated GaAs cones lying flat on a $SiO_2$ substrate and illuminated by a source with a Gaussian beam profile. The dimensions of individual NWs were measured from SEM images and the orientation of the NW relative to the fixed polarisation of the excitation source noted.

### Supplementary Note 5 | Description of the emission from wurtzite nanowires

The emission spectrum of a wurtzite (WZ) GaAs NW as presented in Fig. 1e of the main text is seen to peak at approximately 1.435 eV (865 nm) with a full width at half-maximum (FWHM) of 44 meV. Some confusion remains in the literature regarding the expected peak position of WZ GaAs,[19] and the value found here sits between those currently published, being 10 meV greater than[20] one report and 10-25 meV less than[21-24] several others. We note that unlike the majority of samples characterised elsewhere,[20, 21, 23] our NWs are unpassivated and therefore required relatively high pump powers. Considering the power dependant emission of a WZ GaAs nanowire as presented in Fig. 3b of the main text, a higher energy peak appears at 1.531 eV (810 nm) for photoinjected carrier densities greater than $3x10^{17}$ $cm^{-3}$. This second peak may be identified as the conduction band to light-hole transition[22] and its appearance is a result of photoinduced band-filling as suggested by the

broadening of emission towards higher energy at increased pump powers. Both peaks are seen to slightly redshift with increasing excitation.

### Supplementary Note 6 | Role of NW areal density.

The areal density of NWs was observed to strongly affect both their structure and morphology. Supplementary Fig. 8a-c presents a progressive dilution of a 30 nm colloid used to seed growth, with Supplementary Fig. 8b showing a 25 times dilution relative to Supplementary Fig. 8a and Supplementary Fig. 8c a 250 times dilution. As areal density is reduced, NW height increases and the sidewall morphology is seen to change. Examining the TEM images in Supplementary Fig. 9 the changing appearance of the NW sidewalls can be linked to the introduction of a high density of planar defects. Both the increase in NW height and introduction of planar defects may be considered a product of increased group III supply stemming from the increase in collection area with reduced NW areal density. Where growth is group III limited, an increase in supply will increase the growth rate but also likely the supersaturation and effective V/III ratio, factors both known to affect crystal structure.[25, 26]

One interesting feature of the NWs grown at lower area densities Supplementary Fig. 8b,c,d is the appearance of a tapered segment near the tip of the NWs. This is likely related to a gradual depletion of Ga from the Au seed particle during the cooling phase which was conducted under arsine.[27]

In NWs grown at lower areal densities, a variation in crystal structure was often evident along the length of the NW. Planar defects tended to have a higher density towards the base of the NWs which in some instances gave way to periodic twinning towards the tip of the NWs (see Supplementary Fig. 10). This variation can be related to changes in group III supply as the NW grows in height and its collection area shifts from the substrate to the NW sidewalls.[28] Interestingly, periodic twinning in these NWs grown at low density often showed overgrowth from {111} to {110} type sidewalls.

## Supplementary Note 7 | Numerical modelling of recombination with surface band-bending

Unpassivated GaAs is known to be characterised by a high concentration of surface trap states leading to surface band-bending and depletion.[29-31] These effects have furthermore been reported to significantly affect emission from unpassivated GaAs NWs[9] and may be expected to vary with optical excitation. In order to assess the potential relevance of surface band-bending to our experiments we performed finite volume modelling using the commercial software package *COMSOL Multiphysics*. In an approach previously reported,[32] we solved a system of three coupled differential equations in 1D to give electric potential, hole concentration and electron concentration as a function of radial position. Steady state, spatially uniform carrier generation was defined with losses to surface, bulk SRH, radiative and Auger recombination. Relevant model parameters are given in Supplementary Table 1.

Although the use of steady state conditions represents somewhat of a simplification, we expect the results derived here to hold qualitative relevance to our pulsed excitation experiments. For that reason we considered photoexcitation levels beyond which would normally be accessed by a continuous-wave source in order to include photo-generated carrier concentrations similar to the peak values obtained with pulsed excitation. Indeed, the plot of IQE versus pump power derived here (Supplementary Fig. 13) shows qualitative similarity to that derived from an analytical model (see Supplementary Note 8 and specifically Supplementary Fig. 16). We further note that the energy distribution and density of surface trap states on GaAs surfaces is relatively difficult to measure and will vary with orientation,[31, 33, 34] treatment[35-38] and doping[33, 39-44] and as such a full quantitative treatment should be considered beyond the scope of this paper. Our results here thus represent a qualitative assessment of how the spatial extent of surface band-bending is reduced by both doping and photoexcitation. They suggest that for the high doping densities or high photoexcitation levels that we fit in Fig. 3 of the main manuscript, surface band-bending may be safely neglected. It is also important to note that a reduced surface state density has previously been associated with p-type GaAs,[42] which would act to further reduce the effects of surface Fermi-level pinning for doped NWs.

Supplementary Fig. 11 plots modelled carrier concentrations along the radial direction for increasing acceptor concentrations from left to right and increasing excitation powers from top to bottom. At the lowest of doping densities and excitation levels (Supplementary Fig. 11a), carrier concentration is seen to vary across the entire radial profile indicating full depletion. With increasing excitation the depletion width of the minority carriers (electrons) is firstly seen to reduce (Supplementary Fig. 11d), before at the highest of pump powers the electron and hole concentrations are seen to be both equal and unvarying with radial position (Supplementary Fig. 11j). At a higher doping density,

depletion is observed to be only partial even at low excitation (Supplementary Fig. 11b). Interestingly, in some specific cases of partial depletion, the superposition of carrier diffusion to the surface and surface band bending was seen to generate a minimum (maximum) in electron (hole) concentration near the NW surface (Supplementary Fig. 11e). At the highest of doping densities assessed, surface band bending was negligible even at the lowest of excitation powers (Supplementary Fig. 11c). Considering Supplementary Fig. 11 in its entirety, it is apparent that both heavy doping (right of figure) and high excitation (bottom of figure) act to reduce the spatial extent of band bending.

In order to visualise the spatial extent of surface band bending, we defined a threshold electric field strength (0.1 kV $cm^{-1}$) below which material was considered to be unaffected by surface depletion. The distance from the surface at which the electric field first reached this threshold was considered the 'depletion width'. Supplementary Fig. 12 plots depletion width as a function of excitation for various doping densities. As determined from Supplementary Fig. 11a, at the lowest of doping densities, $2.8x10^{16}$ $cm^{-3}$, surface depletion extends to the centre of the NW which may be considered fully depleted. At a pump density of approximately $1x10^{28}$ $cm^{-3}$ $s^{-1}$ this depletion width is seen to fall dramatically before approaching zero. At this point surface band-bending can be considered screened by the photoexcited carrier concentration.

While higher doping densities give smaller depletion widths at low excitation (around 5 nm for $2.8x10^{19}$ $cm^{-3}$), a reduction in depletion width does not occur until higher excitation levels due to the stronger electric field strengths associated with the space charge region of heavily doped materials. The x-axis at the top of Supplementary Fig. 12 relates the photoexcited carrier densities used in modelling to equivalent powers of a 522 nm 3 μm spot-size CW pump laser. In most cases depletion width is not seen to alter significantly until pump powers of approximately 1 mW suggesting that depletion will be significant across the usual power ranges accessed by CW pumping.

Supplementary Fig. 13 plots IQE, as defined by the rate of radiative recombination divided by the carrier generation rate, as a function of photoexcitation for various doping concentrations. As is similarly observed from the analytical modelling plotted in Supplementary Fig. 16, IQE is seen to be constant at low excitation before beginning to increase beyond a given threshold excitation level towards a peak value at extremely high excitation. This behaviour may be related to magnitude of the photoexcited carrier density relative to the background doping density.

Where the photoexcited carrier density is less than the background carrier density, both non-radiative and radiative recombination increase linearly with excitation density. Beyond this, as the

majority carrier concentration is increased by photoexcitation, radiative recombination increases as the square of photoexcitation. The threshold photoexcitation density in this case may be approximated by the background doping density divided by the minority carrier lifetime. At the highest of photoexcitation densities, Auger recombination begins to dominate and radiative efficiency is reduced.

The greatest contrast between the results presented here and those derived from the rate equation analysis, as presented in Supplementary Fig. 16, is the magnitude of the IQE advantage enjoyed by doped NWs at low photoexcitation densities. Whereas the rate equation analysis finds heavily doped NWs ($4x10^{19}$ $cm^{-3}$) to be up to 3 orders of magnitude more efficient than undoped NWs ($1x10^{16}$ $cm^{-3}$) at low excitation, the modelling here finds an IQE advantage closer to 11 orders of magnitude. This large discrepancy stems from the significant carrier depletion found by the present modelling at lower doping densities (see Supplementary Fig. 11a). Carrier depletion acts to reduce IQE by reducing the rate of radiative recombination more quickly than the rate of non-radiative recombination. Lower equilibrium carrier concentrations also, however, reduce the pump power required for the photoexcited carrier concentration to exceed the equilibrium carrier concentration and produce an increase in IQE. Considering the x-axis at the top of Supplementary Fig. 13 we can thus observe that for 1 μW CW excitation the difference in radiative efficiency reduces to around 5 orders of magnitude. At powers beyond this the two models converge as the depletion width approaches zero.

To better understand the conditions under which surface depletion may be neglected, we repeated the current steady state modelling for a surface trap density of zero ($N_t$=0 $cm^{-2}$). This scenario is effectively equivalent to simple rate equation modelling as the SRV is maintained at $2.2x10^6$ cm $s^{-1}$ but there is no band-bending or carrier depletion. Supplementary Fig. 14 plots the results for both a trap density of 0 and $1x10^{12}$ $cm^{-2}$. For the lowest doping density of $2.8x10^{16}$ $cm^{-3}$, the inclusion of surface trapping is seen to reduce IQE by around 8 orders of magnitude at low pump powers. The two results however converge above an equivalent CW pump power of 1 mW. For the highest doping density of 2.8 $x10^{19}$ $cm^{-3}$, both results are seen to be similar across all pump powers but only fully converge at a higher rate of photoexcitation relative to the undoped case. The effect of surface depletion on IQE can thus be considered negligible for high excitation intensities or high doping concentrations. As all our experimental data fulfils these conditions, it may be modelled using a more straightforward rate equation approach.

**Supplementary Note 8 | Rate equation analysis**

## Describing recombination

As the pulse duration (approximately 300 fs) in our experiments was significantly shorter than the decay process, and the interval between pulses (50 ns) significantly longer, we considered recombination as decay from an initial excited carrier concentration, $N_0$, by non-radiative, radiative and Auger loss terms:

$$\frac{dN}{dt} = -AN - BN(N + N_\mathrm{A}) - CN(N + N_\mathrm{A})^2 \quad (1)$$

where $N$ is the time dependent carrier density, $N_\mathrm{A}$ is the ionised acceptor density and $A$, $B$ and $C$ are the non-radiative, radiative and Auger recombination coefficients respectively. We included an Auger recombination term as some of our experiments employed high pump powers and/or high doping densities. Solving Supplementary Equation 1 numerically for a given photoexcited carrier concentration, $N_0$, and acceptor concentration, $N_\mathrm{A}$, gives IQE through the following integration:

$$\eta_\mathrm{IQE} = \frac{\int BN(t)(N(t) + N_\mathrm{A})dt}{N_0} \quad (2)$$

Given the nanoscale dimensions of our emitters we further took self-absorption to be negligible and equated Supplementary Equation 2 with EQE.

## Determination of the surface recombination velocity

As surface recombination is assumed to be the dominant form of carrier recombination for unpassivated GaAs NWs,[45] the minority carrier lifetime $\tau_\mathrm{mc}$ can be related to the non-radiative recombination coefficient $A$, the surface recombination velocity $S$ and the NW diameter $D$ in the following manner[46]:

$$\frac{1}{\tau_\mathrm{mc}} \cong A \cong \frac{4S}{D} \quad (3)$$

Considering the up-conversion data for the 300 nm diameter doped GaAs NWs presented in Fig. 2 of the main manuscript, a minority carrier lifetime of 3.44 ps thus equates to a non-radiative recombination coefficient of $2.9 \times 10^{11}$ $s^{-1}$ and a surface recombination velocity of $2.18 \times 10^{6}$ cm $s^{-1}$.

## Variation of B and C with doping

Both the radiative and Auger recombination coefficients, $B$ and $C$, are known to vary with dopant concentration.[1, 47] In our modelling we defined these parameters as a function of dopant density on the basis of published experimental results. In the case of the radiative recombination coefficient $B$, its variation with doping in p-type GaAs was investigated by Nelson and Sobers.[1] We fitted the following relationship to their data (see Supplementary Fig. 15):

$$B = -3.47 \times 10^{-11} \ln N_{\mathrm{A}}\,\mathrm{cm^3 s^{-1}} + 1.63 \times 10^{-9}\mathrm{cm^3 s^{-1}} \quad (4)$$

The Auger recombination coefficient is less well defined for GaAs with a range of values having been reported.[48-50] For p-type doping, Ahrenkiel *et al.*[47] found the following relationship:

$$C = 3.83 \times 10^{-43} {N_{\mathrm{A}}}^{0.78}\mathrm{cm^6 s^{-1}} \quad (5)$$

For doping levels of below 1.9x$10^{18}$ $cm^{-3}$, where the above relationship intersects with the value reported by Strauss *et al.*[48] for intrinsic GaAs (7x$10^{-30}$ $cm^{-3}$), we took $C$ to be constant.

## Trends in IQE with doping

Supplementary Fig. 16 plots IQE as modelled through Supplementary Equations 1 and 2 for GaAs NWs with a diameter of 300 nm and a surface recombination velocity of 2.2x$10^{6}$ cm $s^{-1}$. Each curve represents a different level of doping and at low photoexcitation it is apparent that IQE increases with doping from around 0.001% for doping levels of $10^{16}$ $cm^{-3}$ to a peak of 1% for doping levels of 3.6 x$10^{19}$ $cm^{-3}$.

The increase in IQE with doping may be attributed to the second term of the differential equation governing recombination, $BN(N + N_{\mathrm{A}})$, and represents a decreasing radiative lifetime with doping. While $N_{\mathrm{A}} \gg N$, IQE remains constant as the rates of non-radiative, $(\cong AN)$, and radiative, $(\cong BN_{\mathrm{A}}N)$, recombination both increase linearly with photoexcited carrier concentration. Beyond these pump powers, the rate of radiative recombination begins to increase as the square of the photoexcited carrier concentration, $(\cong BN^2)$, and IQE increases steadily with excitation.

At the highest of pump powers Auger recombination becomes significant and IQE is reduced. The threshold for this efficiency droop shifts to lower pump powers with increasing doping but is only significant at extremely high photoexcited carrier concentrations, >$10^{20}$ $cm^{-3}$; well beyond those we achieved experimentally.

Although peak efficiency is highest (≈9%) for the lowest of doping concentrations, $10^{16}$ $cm^{-3}$, the IQE of this NW does not exceed the IQE of a NW doped to 3.6 x$10^{19}$ $cm^{-3}$ until photoexcited carrier

concentrations in excess of 2x10$^{19}$ /cm$^{3}$, a regime where thermal effects are likely to be significant. Importantly, the increase in IQE with doping at low excitation shows a peak at around 3.6 x10$^{19}$ cm$^{-3}$ beyond which Auger recombination becomes significant. The IQE of a NW doped to 1x10$^{20}$ cm$^{-3}$ is seen to be less than that of the NW doped to 3.6x10$^{19}$ cm$^{-3}$ for all pump powers.

### Trends in carrier lifetime with doping

As the equation governing recombination is non-linear, we defined the modelled minority carrier lifetime as the time to achieve a carrier concentration of $N_0/e$. Supplementary Fig. 17 plots this lifetime again for GaAs NWs with a diameter of 300 nm and a surface recombination velocity of 2.2x10$^{6}$ cm s$^{-1}$. At lower pump powers the results appear constant (≈3.5 ps) across the various dopant concentrations which is a consequence of recombination being surface recombination limited. At the highest of doping levels and/or excitation levels, Auger recombination becomes significant and carrier lifetimes are reduced.

### Supplementary Note 9 | Diameter dependence of EQE

The minority carrier lifetime may be defined as follows:

$$\frac{1}{\tau_{\mathrm{mc}}} = \frac{1}{\tau_{\mathrm{nonrad}}} + \frac{1}{\tau_{\mathrm{rad}}} \quad (6)$$

where $\tau_{\mathrm{nonrad}}$ is the non-radiative lifetime and $\tau_{\mathrm{rad}}$ is the radiative lifetime. In our case, surface recombination is the dominant form of recombination and $1/\tau_{\mathrm{nonrad}} \approx 1/\tau_{\mathrm{mc}}$. A similar approximation of Equation 3 from the main manuscript gives $\eta_{\mathrm{IQE}} = \tau_{\mathrm{nonrad}}/\tau_{\mathrm{rad}}$ . Substituting these approximations into Equation 1 of the main manuscript produces:

$$\eta_{\mathrm{IQE}} = \frac{D}{4S} * \frac{1}{\tau_{\mathrm{rad}}} \quad (7)$$

Where surface recombination dominates, EQE is thus expected to be a linear function of nanowire diameter and inversely proportional to both SRV and $\tau_{\mathrm{rad}}$. Supplementary Fig. 18 plots experimentally determined values of EQE for both doped and undoped NWs obtained at a photoexcitation carrier density of 9x10$^{16}$ cm$^{-3}$. A fit of Supplementary Equation 7 gives the parameters $N_{\mathrm{A}}$ = 5x10$^{18}$ cm$^{-3}$ and SRV = 1.5x10$^{6}$ cm s$^{-1}$ for the doped NWs and $N_{\mathrm{A}}$ = 3x10$^{16}$ cm$^{-3}$ and SRV = 1.0x10$^{6}$ cm s$^{-1}$ for the undoped NWs. Deviation of the doped NW dataset towards a more superlinear relationship may be related in this instance to a variation in effective doping density with varying relative shell thickness.

**Supplementary Note 10 | Determining the lasing mode and estimating cavity Q factor**

The threshold gain for the nanowire laser was estimated using the following expression:

$$\Gamma g_{th} \sim \frac{1}{L} \ln \frac{1}{R} \tag{8}$$

where $\Gamma$ is the mode confinement factor, $g_{\text{th}}$is the threshold gain, $L$ is the cavity length and $R$ is the geometric mean of the mode reflectance from each end facet. We performed finite-difference time-domain (FDTD) simulations to calculate $\Gamma$ and $R$ for the guided modes supported in a tapered nanowire lying on $SiO_2$ substrate. The nanowire was modelled as a truncated cone with refractive index of 3.6. The dimensions of the nanowire laser, measured from SEM images, were used for the dimensions of the truncated cone. Only modes that were supported along the entire nanowire, without being cut-off at the narrowest end, were used for these calculations. The mode confinement factor and reflectance were calculated at a fixed wavelength of 880 nm. Since mode confinement varies along the nanowire because of tapering, we calculated $\Gamma$ at the centre of the nanowire and used this value as an estimate.

The threshold gain calculated using Supplementary Equation 8 for the nanowire laser was 3390, 3450, 1140, 2570, 2480, 1050, 1700 and 1600 $cm^{-1}$ for the $HE11_a$, $HE11_b$, TE01, $HE21_a$, $HE21_b$, TM01, $EH11_a$, and $EH11_b$ modes, respectively. The lowest threshold gain was for the TM01 mode, which suggests that the nanowire laser in our experiments was lasing from the TM01 mode. The threshold gain above was estimated at a fixed wavelength. However the lasing spectra shown in the main manuscript has multiple peaks. We used FDTD simulations to verify that these peaks correspond to different axial modes supported in the Fabry-Perot type cavity. In these simulations, we used a dipole source orientated and positioned along the axis of the nanowire to excite TM01 guided modes.[51] As before, the nanowire was modelled as a truncated cone lying on $SiO_2$ substrate, with dimensions corresponding to the measured dimensions of the nanowire laser. The index of the nanowire and substrate were 3.6 and 1.5, respectively. In these simulations, the electric field was monitored at various positions within the nanowire as a function of time. The cavity spectrum was then determined from the Fourier transform of the signal.

Supplementary Fig. 20a shows the simulated cavity spectrum. The peaks in the spectrum correspond to different resonant modes supported in the nanowire. Supplementary Fig. 20b shows the electric field intensity profile in the cross-section of the nanowire at wavelength of 900 nm, corresponding to the spectral position of one of the resonant modes. The field profile in the cross-section transverse to the nanowire axis resembles the profile of the TM01 guided mode and the field profiles in the cross-sections parallel to the nanowire axis show that the resonant mode has an axial order (m) of

33. The mode type and axial order of the resonant modes at other spectral positions was identified from field profiles in a similar way. The axial order of the TM01 resonant modes is denoted above the peaks in Supplementary Fig. 20a. The spectral position of the TM01 resonant modes coincides well with the spectral position of the lasing peaks, as shown in Fig. 4a of the main manuscript. Again this modelling suggests that the nanowire laser characterised experimentally was lasing from the TM01 mode.

The cavity spectrum shown in Supplementary Fig. 20a enables us to estimate the group velocity, or group index of the lasing mode. The wavelength separation between resonant modes in a Fabry-Perot type cavity is given by:

$$\Delta\lambda = \frac{\lambda^2}{2Ln_\mathrm{g}} \tag{9}$$

Where λ is the wavelength, $L$ is the cavity length and $n_\mathrm{g}$ is the group index of the mode. Using Supplementary Equation 9, we estimate $n_\mathrm{g}$ = 4.44 at $\lambda$ = 883 nm for the TM01 mode. The cavity spectrum also enables us to estimate the $Q$ factor for the resonant modes, using $f_\mathrm{R}/\Delta f$, where $f_\mathrm{R}$ is the resonance frequency and $\Delta f$ is the FWHM of the spectral peak. We estimate a $Q$ factor of ~250-350 for the laser cavity from simulations. The $Q$ factor measured from the experimental lasing spectra at threshold is ~300 at λ=883 nm.

## Supplementary Note 11 | Rate equation analysis of lasing

Rate equations were used to fit the experimental L-L curve shown in Fig. 4d of the main manuscript and thereby estimate the doping concentration, $N_\mathrm{A}$, threshold gain, $g_\mathrm{th}$, and spontaneous emission factor, $\beta$. Since there were predominantly three lasing modes in the lasing spectra, multimode rate equations for three cavity modes were used. The rate equations for the carrier density in the active region, $N$, and photon density in the $b^{\mathrm{th}}$ cavity mode, $S_\mathrm{b}$, are as follows:

$$\frac{dN}{dt} = \frac{\eta_\mathrm{p}P}{\hbar\omega V} - AN - BN(N+N_\mathrm{A}) - CN(N+N_\mathrm{A})^2 - \sum_{b=1}^{3} v_\mathrm{g} g S_\mathrm{b}$$
$$\frac{dS_\mathrm{b}}{dt} = \Gamma v_\mathrm{g}(g - g_\mathrm{th})S_\mathrm{b} + \Gamma\beta \cdot BN(N+N_\mathrm{A}) \tag{10}$$

$\eta_\mathrm{p}P/\hbar\omega V$ is the carrier generation rate, where $\eta_\mathrm{p}$, $\hbar\omega$, $P$ and $V$ are the fraction of pump power absorbed, energy of pump photon, optical pump power used and volume of the nanowire, respectively. $\eta_\mathrm{p}$ of ~1% was estimated from FDTD simulations using a Gaussian source with FWHM corresponding to the laser spot size measured from experiments (see Supplementary Fig. 7).

$P$ is a time-dependent function of the form $P_\mathrm{p}\,\mathrm{sech}^2(1.76t/\Delta t)$, where $P_\mathrm{p}$ is the peak power of the pulse and $\Delta t$ = 400 fs is the pulse width. $P_\mathrm{p}$ was calculated from the average power of the pump laser using $P_\mathrm{p}\Delta t = P_\mathrm{ave}/f_\mathrm{p}$, where $f_\mathrm{p}$= 20.8 MHz is the frequency of the pulsed laser. The volume of the nanowire was calculated using $V = \frac{\pi L}{12}(d_1^2 + d_2^2 + d_1 d_2)$, where $d_1 = 400$ nm, $d_2 = 600$ nm and $L = 5.15$ μm are the measured dimensions of the nanowire laser.

The $B$ and $C$ coefficients were defined previously in Supplementary Note 8. $A$ is estimated using Supplementary Equation 3, using a surface recombination velocity of 1.875x$10^6$ cm $s^{-1}$. $B$ and $C$ coefficients are dependent on the injected carrier/doping concentration, and are expressed as functions of $N + N_\mathrm{A}$ using Supplementary Equations 4 and 5, respectively. The material gain, $g$, also depends on the doping/injected carrier concentration.

We modelled the material gain for GaAs at different doping concentrations as described in Supplementary Note 12 and used a logarithmic model for the gain function: $g(N) = g_0 \ln(N + N_\mathrm{s}/N_\mathrm{tr} + N_\mathrm{s})$, where $g_0$, $N_\mathrm{tr}$ and $N_\mathrm{s}$ are parameters determined by curve fitting. The gain function parameters for different doping concentrations are shown in Supplementary Table 2. Note that the material gain is modelled at the centre lasing mode wavelength, 883 nm, and at a temperature of 380 K, which is temperature of the optically pumped nanowire laser.

The temperature and lineshape broadening parameter ($\gamma$=18.8 meV) were estimated by modelling the spontaneous emission spectrum and fitting its shape with the photoluminescence spectrum of the nanowire laser.

Lastly, $v_\mathrm{g}$ and $\Gamma$ are the group velocity and mode confinement factor of the lasing mode, respectively. These parameters are dependent on the lasing mode type and mode wavelength. Here we assume that these parameters are equal for each of the three lasing modes, and use the values of $v_\mathrm{g} = c/4.44$ and $\Gamma = 1.2$ calculated for the TM01 mode at 883 nm (see Supplementary Note 10). We also assume that parameters $g_\mathrm{th}$ and $\beta$ are the same for each of the three lasing modes.

The rate equations in Supplementary Equation 10 were solved for the duration of one duty cycle of the pump laser with initial estimates for $N_\mathrm{A}$, $g_\mathrm{th}$ and $\beta$. The total photon density ($S(t) = \sum_\mathrm{b} S_\mathrm{b}$) was then evaluated and the average photon density was calculated by integrating over time and dividing by the time span. The normalised average photon density as a function of the average pump power ($P$) is shown on a log-log scale in Supplementary Fig. 21, together with the experimental data.

The four curves from the rate equation modelling are for different doping concentrations: $N_\mathrm{A}$ = 0.1, 1, 2 and 5 $\times 10^{19}$ $\mathrm{cm}^{-3}$. The threshold gain ($g_\mathrm{th}$= 1300 $\mathrm{cm}^{-1}$) and spontaneous emission factor ($\beta$ =

0.015) are the same for each of these curves. The threshold gain of 1300 cm$^{-1}$ is close to the threshold gain estimated for the TM01 mode (see Supplementary Note 12). The $\beta$ factor of 0.015 is consistent with the beta factor estimated for other nanowire lasers of similar dimensions.[3] The curve that best fits the experimental data is for $N_{\mathrm{A}}$ = 2×10$^{19}$ cm$^{-3}$.

The photon density evaluated from the rate equations is converted to output power ($P_{\mathrm{out}}$) using the following equation:

$$P_{\mathrm{out}} = \frac{hc}{\lambda} \cdot \frac{\int_0^T S(t)dt}{T} \cdot V_{\mathrm{p}} \cdot v_{\mathrm{g}} \alpha_{\mathrm{m}} \tag{11}$$

where $hc/\lambda$ is the energy of photon, $\int_0^T S(t)dt/T$ is the time averaged photon density, where $T$ is inverse of the pump pulse frequency, $V_{\mathrm{p}} = V/\Gamma$ is the mode volume and $v_{\mathrm{g}}\alpha_{\mathrm{m}}$ is the escape rate of photons. We use the values of $v_{\mathrm{g}}$, $\Gamma$ and $\alpha_{\mathrm{m}} = L^{-1} \ln R^{-1}$ calculated from FDTD modelling (see Supplementary Note 10).

The L-L curve with absolute power units on both axes is shown in Fig. 4e of the main text. The slope of the curve above threshold is about 0.2%. Since the fraction of power absorbed in the nanowire is only about $\eta_p \sim$ 1% of the input power (see above), the absolute slope efficiency (external efficiency) of our laser is ~20%.

## Supplementary Note 12 | Modelling of gain in doped GaAs

Optical gain in a direct band gap bulk semiconductor can be modelled using the following equation:

$$\begin{gathered} g(\hbar\omega) = \frac{\pi e^2}{n_{\mathrm{r}} c \varepsilon_0 m_0^2 \omega} |M|^2 \int \rho_{\mathrm{r}}(E)\big(f_{\mathrm{c}}(E) - f_{\mathrm{v}}(E)\big)\, \ell(E - \hbar\omega)\, dE \\ \ell(E - \hbar\omega) = \frac{1}{\pi\gamma} \operatorname{sech}\left(\frac{E - \hbar\omega}{\gamma}\right) \end{gathered} \tag{12}$$

where $e$, $n_{\mathrm{r}}$, $c$, $\varepsilon_0$, $m_0$, $\hbar\omega$ and $|M|^2$ are electron charge, refractive index, vacuum speed of light, vacuum permittivity, electron mass, photon energy and the momentum matrix element. In the integrand, $\rho_{\mathrm{r}}(E)$ is the 3D reduced density of states function, $f_{\mathrm{c,v}}(E)$ is the Fermi-Dirac distribution for conduction/valance band and $\ell(E - \hbar\omega)$ is the lineshape broadening function which accounts for the energy broadening of electron-hole states. Here we have used a sech lineshape function to avoid unphysical absorption below the band gap, and $\gamma$ is a parameter with dimensions of energy.

Supplementary Equation 12 models the material gain spectrum at a specified carrier density. We use Supplementary Equation 12 to calculate the gain spectrum for doped and undoped GaAs, at

different injection levels. The material parameters used for the calculation are listed in Supplementary Table 3.

For a doped material, we first evaluate the intrinsic carrier concentrations using the charge neutrality condition: $n_0 + N_A^- = p_0 + N_D^+$, where $n_0$ and $p_0$ are the intrinsic electron and hole concentrations and $N_A^-$ and $N_D^+$ are the ionised acceptor and donor concentrations. Note that $n_0 = p_0$ for an undoped material. The intrinsic electron, hole and ionised dopant concentrations are calculated at the specified temperature using Fermi-Dirac statistics. Then we calculate the quasi Fermi-levels at a given injected carrier density, i.e. at electron concentration of $n + n_0$ and hole concentration of $p + p_0$, where $n$ and $p$ are the injected electron and hole concentrations. Note that $n = p$, since optical pumping methods are used in this study. We will refer to the number of injected electron-hole pairs as $N$.

Supplementary Fig. 22a shows the peak material gain as a function of injected carrier density for doped and undoped GaAs. For the doped material, we have modelled the gain for p-type doping concentrations of $10^{18}$ and $10^{19}$ $cm^{-3}$. The material gain for p-type doped GaAs is much larger than for undoped GaAs. This is because p-type doping results in a downward shift in the quasi-Fermi levels, which consequently reduces the transparency carrier density required to achieve gain.[52] Therefore doping can reduce the injected carrier density, or equivalently the optical pump power, required to achieve threshold gain. In addition, p-type also increases the differential gain, because of the alignment of the quasi-Fermi levels with the band edges.[52] The differential gain $dg/dN$ for doped and undoped GaAs is shown in Supplementary Fig. 22b. Large differential gain is required for high speed modulation applications.

## Supplementary References


1. Nelson RJ, Sobers RG. Minority-carrier lifetimes and internal quantum efficiency of surface-free GaAs. *J Appl Phys* **49**, 6103-6108 (1978).
2. Jiang N*, et al.* Long minority carrier lifetime in Au-catalyzed GaAs/AlxGa1−xAs core-shell nanowires. *Appl Phys Lett* **101**, - (2012).
3. Saxena D*, et al.* Optically pumped room-temperature GaAs nanowire lasers. *Nat Photon* **7**, 963-968 (2013).
4. Ettenberg M, Nuese CJ. Comparison of Zn-doped GaAs layers prepared by liquid-phase and vapor-phase techniques, including diffusion lengths and photoluminescence. *J Appl Phys* **46**, 3500-3508 (1975).
5. Casey HC, Stern F. Concentration-dependent absorption and spontaneous emission of heavily doped GaAs. *J Appl Phys* **47**, 631-643 (1976).
6. Lu ZH, Hanna MC, Majerfeld A. Determination of band gap narrowing and hole density for heavily C-doped GaAs by photoluminescence spectroscopy. *Appl Phys Lett* **64**, 88-90 (1994).
7. Montazeri M*, et al.* Transient Rayleigh Scattering: A New Probe of Picosecond Carrier Dynamics in a Single Semiconductor Nanowire. *Nano Letters* **12**, 5389-5395 (2012).

8. Wang Y*, et al.* Carrier Thermalization Dynamics in Single Zincblende and Wurtzite InP Nanowires. *Nano Letters* **14**, 7153-7160 (2014).
9. Demichel O, Heiss M, Bleuse J, Mariette H, Fontcuberta i Morral A. Impact of surfaces on the optical properties of GaAs nanowires. *Appl Phys Lett* **97**, 201907-201903 (2010).
10. Titova LV*, et al.* Temperature dependence of photoluminescence from single core-shell GaAs-AlGaAs nanowires. *Appl Phys Lett* **89**, 73126 (2006).
11. Hoang TB*, et al.* Resonant excitation and imaging of nonequilibrium exciton spins in single core-shell GaAs-AlGaAs nanowires. *Nano Letters* **7**, 588-595 (2007).
12. Mayer G, Maile BE, Germann R, Forchel A, Meier HP. Time resolved spectroscopy on etched GaAs/GaAlAs-quantum-microstructures. *Superlattice Microst* **5**, 579-582 (1989).
13. Izrael A*, et al.* Microfabrication and optical study of reactive ion etched InGaAsP/InP and GaAs/GaAlAs quantum wires. *Appl Phys Lett* **56**, 830-832 (1990).
14. Wang PD, Sotomayor Torres CM, Benisty H, Weisbuch C, Beaumont SP. Radiative recombination in GaAs-AlxGa1−xAs quantum dots. *Appl Phys Lett* **61**, 946-948 (1992).
15. Clausen EM*, et al.* Determination of nonradiative surface layer thickness in quantum dots etched from single quantum well GaAs/AlGaAs. *Appl Phys Lett* **55**, 1427-1429 (1989).
16. Ogawa K, Haraguchi K-i, Hiruma K, Fujisaki Y, Katsuyama T, Fasol G. Spectral and temporal features of photoluminescence of gallium arsenide quantum-wire crystals. *J Lumin* **53**, 387-390 (1992).
17. Henry CH, Lang DV. Nonradiative capture and recombination by multiphonon emission in GaAs and GaP. *Physical Review B* **15**, 989-1016 (1977).
18. Henry C. Deep level spectroscopy, low temperature defect motion and nonradiative recombination in GaAs and GaP. *J Electron Mater* **4**, 1037-1052 (1975).
19. Faustino M, Giacomo P, Silvia R. Photoluminescence of GaAs nanowires at an energy larger than the zincblende band-gap: dependence on growth parameters. *Semiconductor Science and Technology* **30**, 055020 (2015).
20. Moewe M, Chuang LC, Crankshaw S, Chase C, Chang-Hasnain C. Atomically sharp catalyst-free wurtzite GaAs⁄AlGaAs nanoneedles grown on silicon. *Appl Phys Lett* **93**, 023116 (2008).
21. Ahtapodov L*, et al.* A Story Told by a Single Nanowire: Optical Properties of Wurtzite GaAs. *Nano Letters* **12**, 6090-6095 (2012).
22. Kusch P, Breuer S, Ramsteiner M, Geelhaar L, Riechert H, Reich S. Band gap of wurtzite GaAs: A resonant Raman study. *Physical Review B* **86**, 075317 (2012).
23. Signorello G*, et al.* Inducing a direct-to-pseudodirect bandgap transition in wurtzite GaAs nanowires with uniaxial stress. *Nat Commun* **5**,  (2014).
24. Ihn S-G, Ryu M-Y, Song J-I. Optical properties of undoped, Be-doped, and Si-doped wurtzite-rich GaAs nanowires grown on Si substrates by molecular beam epitaxy. *Solid State Commun* **150**, 729-733 (2010).
25. Sebastian L, Daniel J, Kimberly AD. Crystal phase control in GaAs nanowires: opposing trends in the Ga- and As-limited growth regimes. *Nanotechnology* **26**, 301001 (2015).
26. Dubrovskii VG, Grecenkov J. Zeldovich Nucleation Rate, Self-Consistency Renormalization, and Crystal Phase of Au-Catalyzed GaAs Nanowires. *Cryst Growth Des* **15**, 340-347 (2015).
27. Dick KA, Bolinsson J, Borg BM, Johansson J. Controlling the Abruptness of Axial Heterojunctions in III–V Nanowires: Beyond the Reservoir Effect. *Nano Letters* **12**, 3200-3206 (2012).
28. Dayeh SA, Soci C, Bao XY, Wang DL. Advances in the synthesis of InAs and GaAs nanowires for electronic applications [Review]. *Nano Today* **4**, 347-358 (2009).
29. Capiod P*, et al.* Band offsets at zincblende-wurtzite GaAs nanowire sidewall surfaces. *Appl Phys Lett* **103**, 122104 (2013).
30. Chang GS, Hwang WC, Wang YC, Yang ZP, Hwang JS. Determination of surface state density for GaAs and InAlAs by room temperature photoreflectance. *J Appl Phys* **86**, 1765-1767 (1999).

31. Kazuyuki H, Takao N, Akihiko U, Masayuki U. Surface States for the GaAs(001) Surfaces Observed by Photoemission Yield Spectroscopy. *Japanese Journal of Applied Physics* **30**, 3741 (1991).
32. Li Z, Wenas YC, Fu L, Mokkapati S, Tan HH, Jagadish C. Influence of Electrical Design on Core-Shell GaAs Nanowire Array Solar Cells. *Ieee J Photovolt* **5**, 854-864 (2015).
33. Grant RW, Waldrop JR, Kowalczyk SP, Kraut EA. Correlation of GaAs surface chemistry and interface Fermi-level position: A single defect model interpretation. *Journal of Vacuum Science & Technology* **19**, 477-480 (1981).
34. Guichar GM, Sebenne CA, Garry GA. Intrinsic and Defect-Induced Surface States of Cleaved GaAs(110). *Phys Rev Lett* **37**, 1158-1161 (1976).
35. Liu D, Zhang T, LaRue RA, Harris JS, Sigmon TW. Deep level transient spectroscopy study of GaAs surface states treated with inorganic sulfides. *Appl Phys Lett* **53**, 1059-1061 (1988).
36. Offsey SD, Woodall JM, Warren AC, Kirchner PD, Chappell TI, Pettit GD. Unpinned (100) GaAs surfaces in air using photochemistry. *Appl Phys Lett* **48**, 475-477 (1986).
37. Ludeke R, Koma A. Electronic surface states on clean and oxygen-exposed GaAs surfaces. *Journal of Vacuum Science & Technology* **13**, 241-247 (1976).
38. Chang CC, Citrin PH, Schwartz B. Chemical preparation of GaAs surfaces and their characterization by Auger electron and x-ray photoemission spectroscopies. *Journal of Vacuum Science & Technology* **14**, 943-952 (1977).
39. Lodha S, Janes DB, Chen N-P. Unpinned interface Fermi-level in Schottky contacts to n-GaAs capped with low-temperature-grown GaAs; experiments and modeling using defect state distributions. *J Appl Phys* **93**, 2772-2779 (2003).
40. Yan D, Look E, Yin X, Pollak FH, Woodall JM. Air stabilized (001) p-type GaAs fabricated by molecular beam epitaxy with reduced surface state density. *Appl Phys Lett* **65**, 186-188 (1994).
41. Yin X*, et al.* Photoreflectance study of the surface Fermi level at (001) n- and p-type GaAs surfaces. *Journal of Vacuum Science & Technology A* **10**, 131-136 (1992).
42. Pollak FH. Contactless electromodulation investigations of surface/interface electric fields in semiconductor microstructures. *J Vac Sci Technol B* **11**, 1710-1716 (1993).
43. Hinkle CL*, et al.* Comparison of n-type and p-type GaAs oxide growth and its effects on frequency dispersion characteristics. *Appl Phys Lett* **93**, 113506 (2008).
44. Jastrzebski L, Lagowski J, Gatos HC. Application of scanning electron microscopy to determination of surface recombination velocity: GaAs. *Appl Phys Lett* **27**, 537-539 (1975).
45. Joyce HJ*, et al.* Electronic properties of GaAs, InAs and InP nanowires studied by terahertz spectroscopy. *Nanotechnology* **24**, 214006 (2013).
46. Leonard F, Talin AA, Swartzentruber BS, Picraux ST. Diameter-Dependent Electronic Transport Properties of Au-Catalyst/Ge-Nanowire Schottky Diodes. *Phys Rev Lett* **102**, 106805 (2009).
47. Ahrenkiel RK, Ellingson R, Metzger W, Lubyshev DI, Liu WK. Auger recombination in heavily carbon-doped GaAs. *Appl Phys Lett* **78**, 1879-1881 (2001).
48. Strauss U, Rühle WW, Köhler K. Auger recombination in intrinsic GaAs. *Appl Phys Lett* **62**, 55-57 (1993).
49. Capizzi M, Modesti S, Frova A, Staehli JL, Guzzi M, Logan RA. Electron-hole plasma in direct-gap $Ga_{1-x}Al_xAs$ and *k*-selection rule. *Physical Review B* **29**, 2028-2035 (1984).
50. Benz G, Conradt R. Auger recombination in GaAs and GaSb. *Physical Review B* **16**, 843-855 (1977).
51. Paniagua-Dominguez R, Grzela G, Rivas JG, Sanchez-Gil JA. Enhanced and directional emission of semiconductor nanowires tailored through leaky/guided modes. *Nanoscale* **5**, 10582-10590 (2013).
52. Coldren LA, Corzine SW, Mashanovitch ML. *Diode lasers and photonic integrated circuits*. John Wiley & Sons (2012).
